# Discovering monotonic stemness marker genes from time-series stem cell microarray data

**DOI:** 10.1186/1471-2164-16-S2-S2

**Published:** 2015-01-21

**Authors:** Hsei-Wei Wang, Hsing-Jen Sun, Ting-Yu Chang, Hung-Hao Lo, Wei-Chung Cheng, George C Tseng, Chin-Teng Lin, Shing-Jyh Chang, Nikhil Ranjan Pal, I-Fang Chung

**Affiliations:** 1 Institute of Biomedical Informatics, National Yang-Ming University, Taipei, Taiwan; 2 Center for Systems and Synthetic Biology, National Yang-Ming University, Taipei, Taiwan; 3 Institute of Microbiology and Immunology, National Yang-Ming University, Taipei, Taiwan; 4 VGH-Yang-Ming Genome Research Center, National Yang-Ming University, Taipei, Taiwan; 5 The Molecular Biology Institute, University of California Los Angeles, LA, CA, USA; 6 Research Center for Tumor Medical Science, China Medical University, Taichung, Taiwan; 7 Department of Biostatistics, University of Pittsburgh, Pittsburgh, PA, USA; 8 Department of Electrical Engineering, National Chiao-Tung University, Hsinchu, Taiwan; 9 Brain Research Center, National Chiao-Tung University, Hsinchu, Taiwan; 10 Department of Obstetrics and Gynecology, Hsinchu Mackay Memorial Hospital, Hsinchu, Taiwan; 11 Electronics and Communication Sciences Unit, Indian Statistical Institute, Calcutta, India

## Abstract

**Background:**

Identification of genes with ascending or descending monotonic expression patterns over time or stages of stem cells is an important issue in time-series microarray data analysis. We propose a method named Monotonic Feature Selector (MFSelector) based on a concept of total discriminating error (DE_total_) to identify monotonic genes. MFSelector considers various time stages in stage order (i.e., Stage One vs. other stages, Stages One and Two vs. remaining stages and so on) and computes DE_total _of each gene. MFSelector can successfully identify genes with monotonic characteristics.

**Results:**

We have demonstrated the effectiveness of MFSelector on two synthetic data sets and two stem cell differentiation data sets: embryonic stem cell neurogenesis (ESCN) and embryonic stem cell vasculogenesis (ESCV) data sets. We have also performed extensive quantitative comparisons of the three monotonic gene selection approaches. Some of the monotonic marker genes such as *OCT4*, *NANOG*, *BLBP*, discovered from the ESCN dataset exhibit consistent behavior with that reported in other studies. The role of monotonic genes found by MFSelector in either stemness or differentiation is validated using information obtained from Gene Ontology analysis and other literature. We justify and demonstrate that descending genes are involved in the proliferation or self-renewal activity of stem cells, while ascending genes are involved in differentiation of stem cells into variant cell lineages.

**Conclusions:**

We have developed a novel system, easy to use even with no pre-existing knowledge, to identify gene sets with monotonic expression patterns in multi-stage as well as in time-series genomics matrices. The case studies on ESCN and ESCV have helped to get a better understanding of stemness and differentiation. The novel monotonic marker genes discovered from a data set are found to exhibit consistent behavior in another independent data set, demonstrating the utility of the proposed method. The MFSelector R function and data sets can be downloaded from: http://microarray.ym.edu.tw/tools/MFSelector/.

## Background

In many biological experiments, we analyze regulation of gene or protein expression over time and in some other cases we examine how the expression pattern changes as the grade/stage of a disease progresses, including over time.

These biomarkers are "Monotonic" because their pattern ascends or descends with time (or stage/grade), important in time-series studies (i.e., disease progression, stem cell differentiation, aging and drug kinetics experiments)[1,2]. For example, HNF4α is a well-known factor promoting stem cell hepatogenesis[3], and its levels increase with a monotonic pattern during differentiation[4]. Genes in such studies may exhibit a natural temporal ordering in the samples, distinguishing them from others dealing with issues like discriminating tumor from non-tumor cases. A time series model generally shows that samples close in time are more closely related. For example, the expression of a stemness gene or microRNA should be more abundant in stem cells and precursors than in fully differentiated and mature progenies. Here we identify those genes monotonically ascending or descending over a given period of time.

Many approaches are used to find differentially expressed biomarkers from microarray data [5-8]. By statistical tests and other computational methods, the two-class and multi-class array data are usually analyzed focusing on the class-specific signature genes (i.e., genes that are expressed for one/some class(es) and unexpressed for the other/rest of the class(es)). In order to identify differentially expressed genes, many approaches adopt Student's t-test for two-class data, and few approaches adopt statistical tests such as ANOVA F-test for multi-class data [9] or a nonparametric rank-based statistical test such as Kruskal-Wallis test [10] also for multi-class data [11-14]. However, distribution-based and rank-based statistical tests may not be appropriate because they find differentially expressed genes, not monotonically ascending or descending genes.

Some methods to discover monotonic expression patterns are correlation-based, where a seed feature or template is chosen to find genes with good correlation. But the choice of the template plays a critical role, and this is time-consuming, requiring experienced bioinformaticians. In addition, the Cuzick-test [15] can be used to discover a specific trend/pattern from microarray data. Also, the modified M statistic test [16] can be used to detect monotonic trend through a combination of the means ordering strategy and a distribution-based statistical test. However, outliers affect the monotonic trend there because of weighting used in the Cuzick-test and the means ordering strategy used in the modified M statistic test. Furthermore, the Cuzick-test does not provide any information about the extent a gene expression pattern is monotonic (i.e., the degree of "monotonicity") for genes with the same *z*-score/*p*-value. Therefore, an approach without statistical assumptions and not affected by outliers, and able to identify the degree of monotonicity between genes with the same *z*-score/*p*-value, is needed. Our method proposed herein, MFSelector (Monotonic Feature Selector) based on the total discriminating error (DE_total_), is quite effective in discovering genes with monotonic attributes. It identifies gradually increasing or decreasing genes irrespective of any heterogeneity in the array data. Furthermore, MFSelector, eliminates the subjectivity required in seed/template selection and also provides assessments of confidence (i.e., *p*-/*q*-value and sample variance for discriminating error). This new approach is user-friendly as we integrate our algorithm into a simple R function. Users with no proficiency in programming can generate a monotonic gene list and related scatter plots.

## Methods

### Data Sets

All microarray data sets were downloaded from the NCBI GEO public archive, generated in Affymetrix Human Genome U133 Plus 2.0 platform with 54675 probe sets on chips. The raw data (CEL file) were normalized by RMA algorithm using the 'affy' package of the Bioconductor http://www.bioconductor.org[17] software suite in the R Project for statistical Computing http://www.r-project.org. See Materials S1 in Additional file 1 for details.

#### Embryonic Stem Cell Neurogenesis data set (ESCN)

This data set contains 27 samples over five periods of human embryonic development: three embryonic stem cell (ESC) samples, three embryoid body (EB) samples, six primitive ectoderm cell (PEL) samples, six neural tube-like rosette cell samples, and nine post-natal neural stem cell (NSC) samples.

#### Embryonic Stem Cell Vasculogenesis data set (ESCV)

In this data set, there are 13 samples over four periods of human embryonic stem cell differentiation into human mature (vascular) endothelial cells: three undifferentiated embryonic stem cell (ESC) samples, three mesodermal progenitor cell (MPC) samples, four embryoid body (EB) samples, and three human mature vascular endothelial cell (VEC) samples. The results and discussion of application of MFSelector to this data set are exhibited in Materials S1 (in Additional file 1).

#### Synthetic data sets

We generated two sets of synthetic data. These data sets contain 50 samples each spread over five equal sized stages. There is a set with descending trends (denoted 'Des') and a set with ascending trends (denoted 'Asc'). These synthetic data sets are named 's50_Asc' and 's50_Des', respectively. In addition, each data set has 120 genes which are classified into 9 types of monotonic genes (or monotonic-like genes): 'Good (distinct)', 'Good (close)', 'Slightly', 'Outliers (slight)', 'Outliers (severe)', 'Moderately', 'Severely', 'Partially ordered (far)', and 'Partially ordered (close)'. There are 20 genes for each of 'Slightly', 'Moderately', and 'Severely' types and 10 genes for each of 'Good (distinct)', 'Good (close)', 'Outliers (slight)', 'Outliers (severe)', 'Partially ordered (far)', and 'Partially ordered (close)'. If most of the samples follow a trend over time/stages but only a few do not, we call those few samples as outliers. In this study the number of outliers for the synthetic data sets is restricted to less than 6% of total samples.

**Figure 1:**
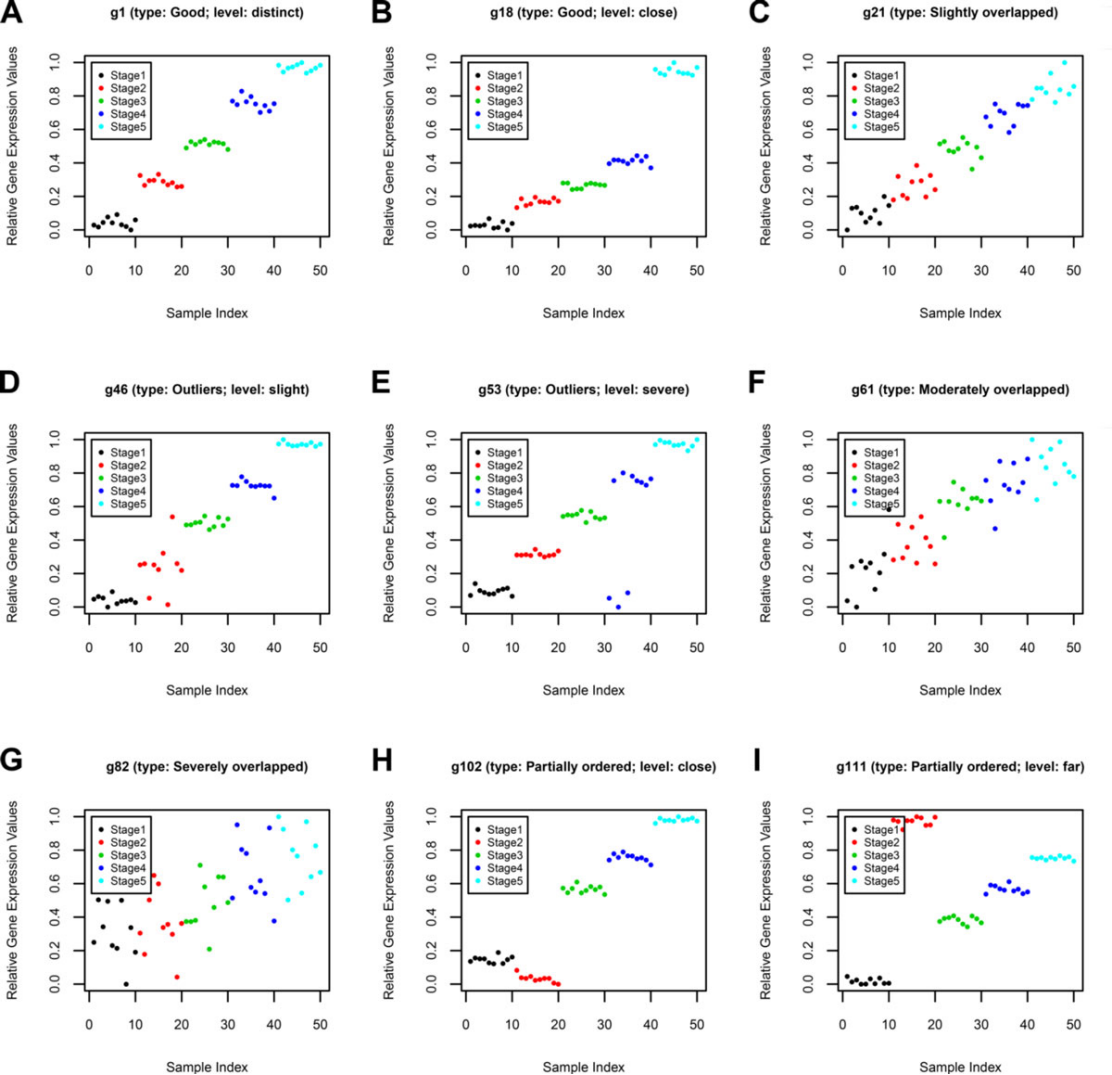
**Scatter plots of nine types of monotonicity**. Examples of each type of genes in the synthetic data set. (A) *g1 *(a 'Good (distinct)' type gene); (B) *g18 *(a 'Good (close)' type gene); (C) *g21 *(a 'Slightly' type gene); (D) *g46 *(a 'Outliers (slight)' type gene); (E) *g53 *(a 'Outliers (severe)' type gene); (F) *g61 *(a 'Moderately' type gene); (G) *g82 *(a 'Severely' type gene); (H) *g102 *(a 'Partially ordered (close)' type gene); (I) *g111 *(a 'Partially ordered (far)' type gene).

Here we use genes with a monotonic ascending trend to illustrate these nine types of genes used in the synthetic data as shown in Figure 1. In this study, genes with a monotonic ascending trend are those where the average expression values of samples in Stage One are lower than those of samples in Stage Two, the average expression values of samples in Stage Two are lower than those of samples in Stage Three, and so on. In the synthetic data sets, 'Good' type genes have the strongest degree of monotonicity followed in order with 'Slightly', 'Moderately', and 'Severely' monotonic type genes. For 'Good' type genes, there is no overlapping of samples between stages, and for 'Severely' type genes, the samples in one stage have samples that severely overlap the samples in the adjacent/other stages. In addition, 'Partially ordered' type genes indicate that the samples in four of the five stages form a monotonic ascending trend, while the samples in the remaining stage overlap the samples in other stages. 'Outliers' type genes denote that samples in the five stages form a monotonic ascending trend, but some of the samples in one of the five stages are slightly/significantly away from the expression values of other samples in the same stage.

The 'Good' category has two subcategories, 'Good (distinct)' and 'Good (close)'. 'Good (distinct)' genes indicate they have the strongest monotonic ascending trend and samples have distinct separation between stages; while 'Good (close)' genes indicate they still have the strongest monotonic trend but samples between stages are close. 'Partially ordered' type genes are also further separated into 'Partially ordered (far)', and 'Partially ordered (close)'. The 'Partially ordered (far)' means that the samples in one of the stages are significantly far from the monotonic trend in a gene expression profile while 'Partially ordered (close)' means that the samples in one of the stages are close to the monotonic trend but it is still out of monotonic order in the gene expression profile. The genes of type 'Outliers (severe)' represent genes where at most six percent of the samples are relatively away from the monotonic trend, and type 'Outliers (slight)' indicates that at most six percent of the samples are slightly away from the monotonic trend. The results on these synthetic data sets by our proposed method (MFSelector) and other two methods are presented in the Results section.

### Monotonic Feature Selector (MFSelector)

**Figure 2:**
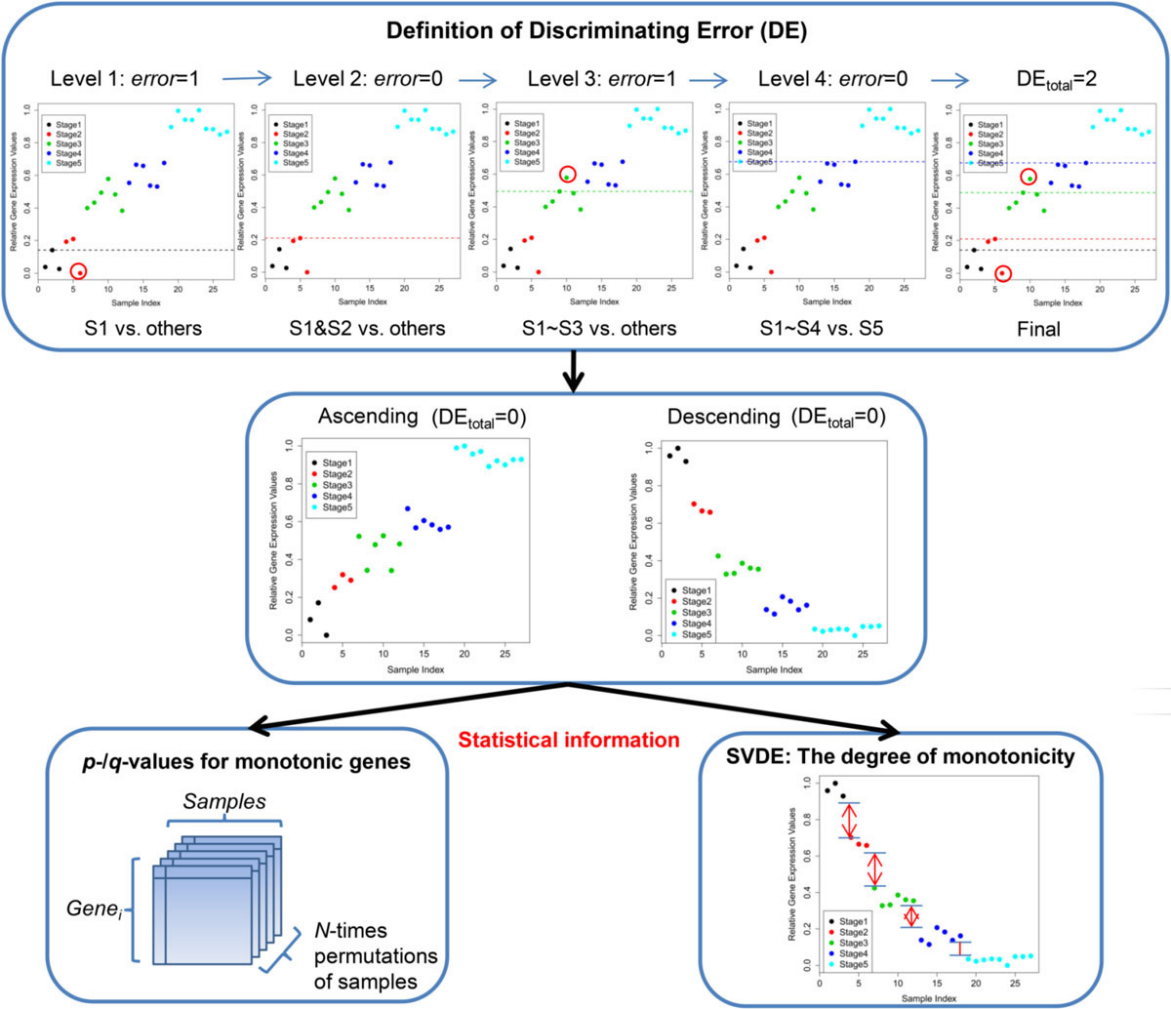
**MFSelector processing steps**. For a gene, the illustration of discriminating error (DE) in each level, total discrimination error (DE_total_), and two statistical analyses is shown in this figure.

In this study, we propose a novel tool, Monotonic Feature Selector (MFSelector) which includes a novel index, called DE_total _(total discriminating error), used to identify genes/biomarkers with stronger monotonic features which may bear a correlation to stem cell development. DE_total _does not make any distributional assumption about the data. MFSelector also provides the related statistical information (*p*- and *q*- value) for each monotonic gene. Moreover, in order to distinguish among monotonic genes with the same DE_total_, MFSelector also offers an additional novel index, called SVDE (sample variance for discriminating error). Users can download MFSelector from the web site: http://microarray.ym.edu.tw/tools/MFSelector/. Figure 2 depicts the processing steps involved in the computation of DE_total_, *p*- and *q*- values, and SVDE.

### Computation of discriminating error for each level of a gene

Here we use several steps to examine genes with monotonically ascending or descending profiles, which are distributed over *N *stages. For example, when a monotonic gene has an ascending profile, first we assume samples in Stage One have the lower expression values and samples in other stages have higher expression values. We use a sample in Stage One to draw a horizontal discriminating line to determine how many samples in Stage One have higher expression values than the discriminating line and how many samples in other stages have lower expression values than the discriminating line. The number of samples on the wrong side of the discriminating line is referred to as "discriminating errors". Distinguishing Stage One from all other stages is called the "Level One" process. In addition, since every sample in Stage One can be used to draw a discriminating line, we select the discriminating line with the smallest number of discriminating errors for this level. Note that, when more than one discriminating line for Stage One have the same number of discriminating errors, we can choose any one of the lines; here we use the discriminating line corresponding to the lowest sample number. Secondly, again for the same gene, as a "Level Two" process, we assume samples in Stages One and Two have lower expression patterns than samples in other stages. Therefore, we use each sample from Stages One and Two taken together to draw a discriminating line and determine discriminating error as in Level One. When more than one sample from Stages One and Two together result in the same smallest number of discriminating errors, we consider: (a) when the samples are from the same stage, then we select the line (hence the sample) corresponding to the lowest sample number; (b) when the samples are from different stages, we give priority to the lowest sample number from the highest stage, in this case, Stage Two. This entire process is repeated *N*-1 times, each time adding one stage (e.g., for the "Level Three" process the union of Stages One, Two and Three versus all other stages; for the "Level Four" process the union of Stages One, Two, Three and Four against all other stages, and so on until the "Level *N*-1"). The discriminating line is selected in the same manner for each of the *N*-1 levels (in total, *N*-1 discriminating lines are considered for a gene). It is interesting to note that, if the number of distinct discriminating lines is equal to *N*-1, the corresponding gene is more likely to be a good monotonic gene.

Our outlier strategy is different from that used by rank-based statistical tests (e.g., Cuzick-test), where an outlier's deviation in expression value influences its rank, often adversely affecting the statistical calculation. MFSelector does not count samples on the wrong side of the discriminating line more than once. Suppose an outlier adds a discriminating error in the Level *M *process. This outlier is very likely to add a discriminating error in later levels, i.e., "Level *M*+1" to "Level *N*-1"). But we count the outlier's discriminating error only once at Level *M*. We compare the results for the Cuzick-test and ours in the Results section.

For a monotonic gene with a descending profile, samples in Stage One should have the higher expression values than the samples in other stages. Thus, we can get the "discriminating errors" for "Level One" process by drawing a discriminating line through one of the samples in Stage One to determine the number of samples in Stage One that have lower expression values than the discriminating line and the number of samples in other stages having higher expression values than the discriminating line. As with an ascending profile, this process is repeated *N*-1 times, though in this case, in descending direction. The discriminating line for each level is also selected in the same manner as that for the ascending case.

### Computation of total Discriminating Error (DE_total_) for a gene

In order to evaluate monotonicity of a gene (e.g., genes with weak or strong monotonic features), we use the sum of its discriminating errors over all *N*-1 level processes (total discriminating error) denoted by "DE_total_". For instance, when DE_total_=*k*, *k *discriminating errors exist over *N*-1 level processes. Here, the smaller DE_total _of a gene, the fewer the number of discriminating errors and the stronger the monotonicity of the gene. DE_total _is an evaluation index to determine monotonic genes. We sort the DE_total _values for all genes in increasing order to select genes with stronger monotonic features for multi-stage and time-series array data. Note that, in this study we separately consider the DE_total _for monotonically ascending or descending cases. In addition, we also develop another strategy using sample variance for discriminating error (SVDE) to further determine the gene with stronger monotonic feature when several genes have the same DE_total _value. This is explained later.

### Calculating DE_total _for a gene: an illustration

**Figure 3:**
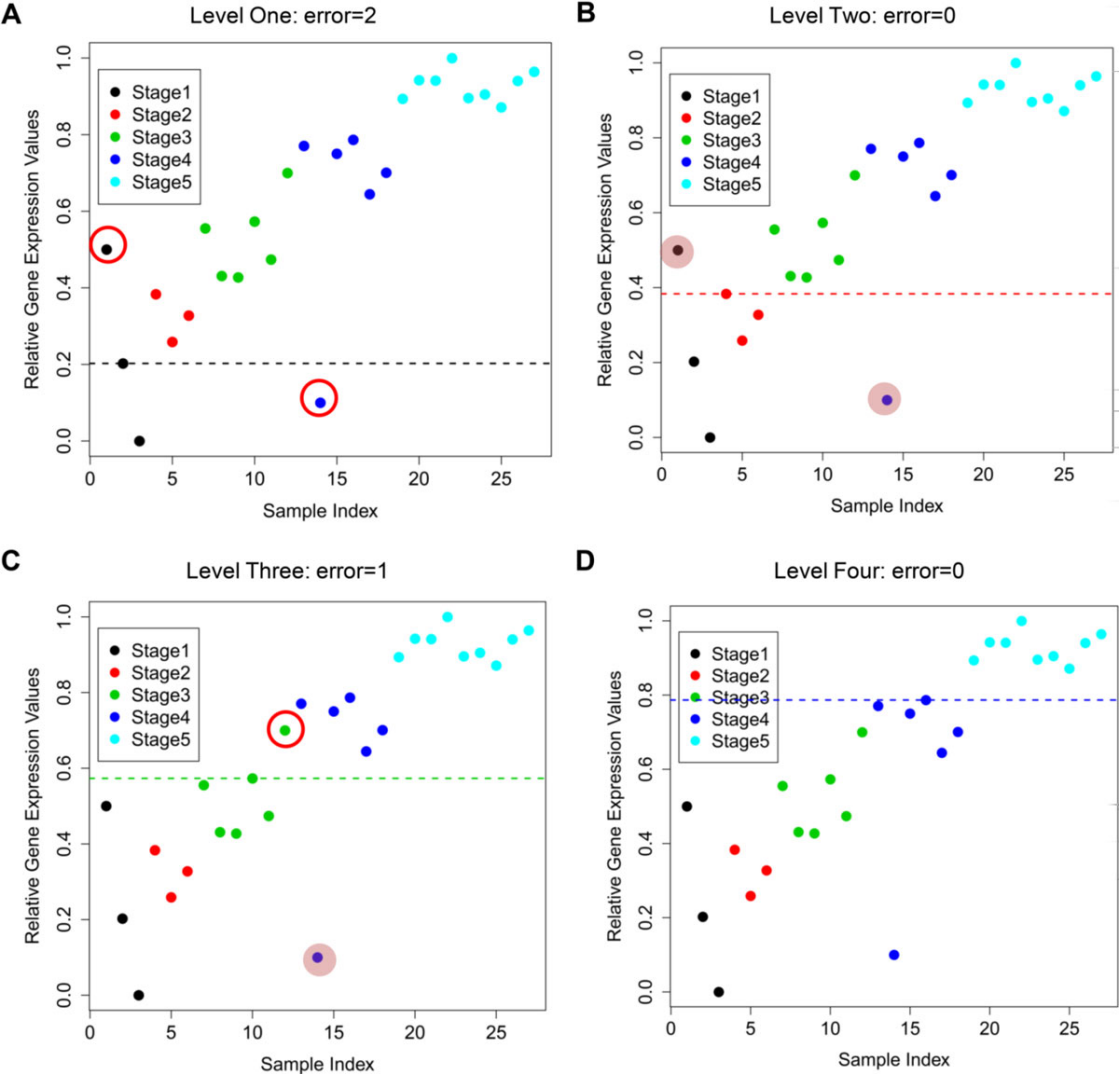
**A synthetic gene profile**. Panels (A) to (D) illustrate the processes of determining discriminating lines and discriminating errors in each stage sequentially. The DE_total _of this synthetic gene is three. The newly identified discriminating errors are marked by red circle and will be counted in a specific stage. The previously identified discriminating errors are indicated by shaded background and will not be counted in a specific stage.

We now demonstrate our algorithm using a synthetic gene profile with five stages as shown in Figure 3. Suppose in this case, this gene has an ascending nature. We perform the following steps for this gene as mentioned above.

First, for the "Level One" process, every sample in Stage One is used to draw a discriminating line to determine its associated number of discriminating errors (the number of samples in Stage One that have higher expression values than the discriminating line and the number of samples in other stages having lower expression values than the discriminating line). In this case, we obtain 7, 2, and 2 discriminating errors taking each of three samples in order from Stage One to draw the discriminating lines. For example, one sample from Stage One and another sample from Stage Four are on the wrong side of the discriminating line when considering the 2^nd ^sample in Stage One to draw the discriminating line, resulting in two discriminating errors. In addition, since two discriminating lines (i.e., corresponding to the 2^nd ^and 3^rd ^samples) from Stage One have the same number of discriminating errors, we choose the sample with the lowest sample number (i.e., 2^nd ^sample) and get two discriminating errors in this level.

Second, for the "Level Two" process, every sample in Stages One and Two is used to draw a discriminating line to determine its associated number of discriminating errors. In this level, we obtain 3, 3, 4, 0, 2, and 1 discriminating errors taking each of six samples in order from Stages One and Two to draw the discriminating lines. Note that, to compute the discriminating errors for the "Level Two" process, we do not consider the samples which have already caused discriminating errors in "Level One". Such samples are masked pink in Figure 3(B), one sample from Stage One and another sample from Stage Four. The extent of outliers may strongly influence results in other methods, but in our method we consider "outliers" only once to contribute to the discriminating errors and hence it allows us to determine monotonicity without the results being skewed by the position of "outliers". This is why our method has some advantage over other approaches. Finally, we choose the sample with the lowest number of discriminating errors (i.e., the 4^th ^sample from Stage One and Two) and get no discriminating errors in this level.

Third, for the "Level Three" process, every sample in Stages One, Two, and Three is used to draw a discriminating line to determine its associated number of discriminating errors. We get 3, 9, 10, 6, 8, 7, 2, 4, 5, 1, 3, and 1 discriminating errors taking each of 12 samples in order from Stage One to Stage Three. The 10^th ^sample is selected as the discriminating line because its discriminating error is the lowest, and it has the lowest sample number of the two Level Three samples with the same discriminating error of one.

Similarly, the process is continued for the "Level Four". In this case we determine the 16^th ^sample with no discriminating error. Finally, we add up all discriminating errors for each selected discriminating line from every level and get the DE_total _= 2 + 0 + 1 + 0 = 3.

### Statistical analysis of monotonic genes

#### Computation of p-value and q-value

To assess the statistical significance of the DE_total _index associated with the identified genes in ascending and descending profiles, a permutation test has been performed in Figure 2. Both unadjusted *p*-values and adjusted *q*-values for multiple comparisons are computed as in our previous studies [7,8]. Let *G *be the total number of genes and *S *be the total number of sample points. The procedure followed is summarized below.

Step 1. Given a gene expression matrix *X *(*x_gs _*is the expression intensity of gene *g *in sample unit *s*; 1 ≤ *g *≤ *G*, 1 ≤ *s *≤ *S*) with class labels (*y_s_*, 1 ≤ *s *≤ *S*), we compute the DE_total,g _for ascending case and denotes it by *a_g _*for notational simplicity. Similarly, for the descending case also we compute DE_total,g _and denote it by *d_g_*.

Step 2. Randomly permute the class labels *y_s _B *times (500 times in this study). In the *b^th ^*permutation (1 ≤ *b *≤ *B*), compute $a_{g}^{\left(b\right)}$ and $d_{g}^{\left(b\right)}$ for gene *g *using the gene expression matrix *X *and the permuted labels $y_{s}^{\left(b\right)}$.

Step 3. The *p*-value of the observed DE_total,g _with ascending characteristic, *a_g_*, for gene *g *is

(1) $$p\left(a_{g}\right)=\frac{\sum_{b=1}^{B}\sum_{g^{\prime }=1}^{G}\left(a_{g^{\prime }}^{\left(b\right)}\leq a_{g}\right)}{G\times B}$$

where *I*(·) is an indicator function that takes the value one when true and takes the value zero when false. Similarly, the *p*-value of the observed DE_total,g _with descending nature, *d_g_*, is

(2) $$p\left(d_{g}\right)=\frac{\sum_{b=1}^{B}\sum_{g^{\prime }=1}^{G}I\left(d_{g^{\prime }}^{\left(b\right)}\leq d_{g}\right)}{G\times B}$$

Step 4. To account for the multiple tests being performed for the *G *genes, *q*-values of the observed *a_g _*and *d_g _*are calculated as

(3) $$q\left(a_{g}\right)=\frac{\sum_{b=1}^{B}\sum_{g^{\prime }=1}^{G}I\left(a_{g^{\prime }}^{\left(b\right)}\leq a_{g}\right)}{\sum_{g^{\prime }=1}^{G}I\left(a_{g^{\prime }}\leq a_{g}\right)\times B}$$

and

(4) $$q\left(d_{g}\right)=\frac{\sum_{b=1}^{B}\sum_{g^{\prime }=1}^{G}I\left(d_{g^{\prime }}^{\left(b\right)}\leq d_{g}\right)}{\sum_{g^{\prime }=1}^{G}I\left(d_{g^{\prime }}\leq d_{g}\right)\times B}$$

#### Computation of sample variance for discriminating error (SVDE)

Statistical tests are unable to distinguish which genes with the same level of statistical significance are better. Two genes with the same DE_total _may exhibit different monotonicities. Therefore, we develop SVDE to address the degree of monotonicity of a gene. This can also help to differentiate between genes with the same DE_total _value.

**Figure 4:**
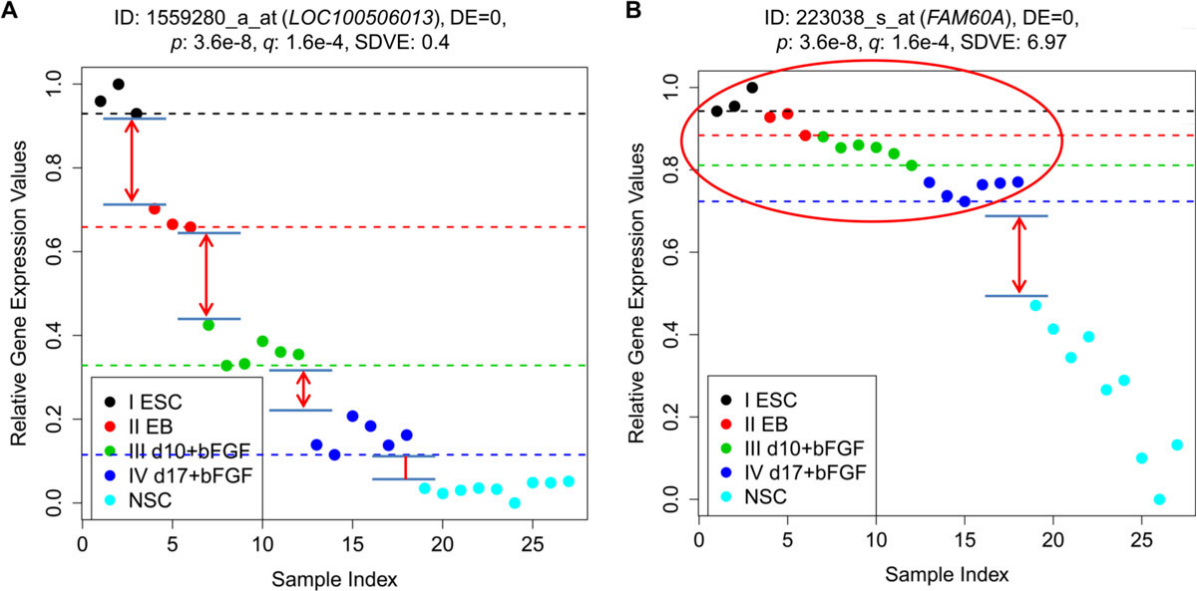
**Scatter plots of 1559280_a_at (*LOC100506013*) and 223038_s_at (*FAM60A*) of the ESCN data set**. Each has a DE_total _equal to zero.

For example, in the ESCN data set, *LOC100506013 *and *FAM60A *are monotonically descending genes and have the same DE_total _= 0 (shown in Figure 4). However, the expressions of the samples of *FAM60A *from Stages One to Four are tightly grouped (albeit not overlapped) and of *LOC100506013 *from Stages One to Four are highly distinguished, and only the expression values of the samples from Stages Four and Five are somewhat close. For these two genes with the same DE_total_, *LOC100506013 *should have a higher degree of monotonicity (Figure 4(A)) than *FAM60A *(Figure 4(B)).

In order to assess the degree of monotonicity (particularly when more than one gene have the same DE_total_), all samples for each of the genes are slightly altered in expression values to examine whether the DE_total _of the altered expression values has changed significantly or not. To evaluate this, we propose an index, called Sample Variance for Discriminating Error (SVDE). We perturb a dataset and evaluate the extent of confidence on the monotonicity properly by adding noise to all samples (as shown in Additional file 2: Fig. S1), and apply the same method to the perturbed a dataset to calculate the DE_total _of genes.

To perturb a dataset with *m *samples and *n *genes, we first compute the standard deviation *σ_i _*of each gene *x_i _*and divide it by 10 to compute *σ'_i_*. Next, we generate *m *noise values from a normal distribution with mean equal to 0 and standard deviation equal to *σ'_i _*for each gene. Finally, we add such a random noise to every observed value of gene *x_i_*. The noise corrupted gene is used to compute its discriminating errors for each level. Let the resulting total discriminating error for this noisy gene be DE_total_. We repeat this procedure in the same manner *M *times (*M *= 100 in this study), and get *M *new DE_total _values for each gene. We denote the new DE_total _value as the DE*_i_*, *i *= 1, 2, ..., *M*. Next we compute the variance of these DE*_i _*values with respect to original DE_total _for this gene. We denote the original DE_total _as DE_org_. Hence for these new DE_total _values we can calculate the variance as

(5) $$SVDE=\frac{1}{M}\overset{M}{\underset{i=1}{\sum }}{\left(DE_{i}-DE_{org}\right)}^{2}$$

The SVDE is a measure of how far the set of the new DE_total _values are spread around the original DE_total _(DE_org_). If each DE*_i _*is equal to or close to DE*_org_*, the SVDE will be very small. Figure 4(A) shows how hard it is to change the DE_total _of a highly confident monotonic gene even after randomly altering the expression values of all samples. The smaller the SVDE, the stronger the monotonicity of the gene. Figure 4(B) depicts the monotonic profile of another gene where DE_total _is equal to zero, but the SVDE is high. The reason is that separation between adjacent stages for Stages One to Four is not sufficiently distinct. Consequently, genes with a smaller SVDE can be considered more strongly monotonic than genes with a larger SVDE.

One may think that the time interval between observations should be given importance in defining any index for finding such stemness markers. This is not so because here our objective is to discover monotonic stemness marker genes which are associated with stages of stem cell differentiation. These stages are clinical stages which depend on pathology/clinical phenotypes and are not defined taking observations with arbitrary/equal time interval, but the biological process here dictates the time interval between observations. However, our method would be equally applicable to other experimental scenario where the researchers design experiments to take observations with equal time interval for some scientific reasons. It does not matter if the required time interval is large or small.

### Cuzick-test

In order to further validate the ability of our method to identify monotonic genes, we have made a comparison with the Cuzick-test [15], an extension of a Wilcoxon-type statistical test for discovering specific patterns with pre-designed trends, such as oscillation, step-wise patterns, and in this case monotonicity. Here we use the Cuzick-test to compute the Cuzick value (z-score) for the gene expression data for each gene as follows. Let *n *be the total number of samples from all classes, *r_i _*be the rank of the *i^th ^*sample determined by the order of its expression value among all samples, *N *be the number of stages, and *p_i _*be the proportion of samples from class *i*. Each stage has a weight *w_k_*, *k *= 1,...,*N*. We associate a weight *z_i _*to a sample *i*, *z_i _*= *w_j_*, if the *i^th ^*sample is from the *j^th ^*stage. The Cuzick statistic is computed as

(6) $$Z=\frac{T-E\left(T\right)}{\sqrt{Var\left(T\right)}}$$

where

(7) $$T=\overset{n}{\underset{i=1}{\sum }}z_{i}r_{i}$$

(8) $$Var\left(T\right)=\left(\frac{n^{2}\left(n+1\right)}{12}\right)\cdot \left(\overset{N}{\underset{i=1}{\sum }}z_{i}^{2}p^{i}-{\left[\overset{N}{\underset{j=1}{\sum }}z_{j}p_{j}\right]}^{2}\right)$$

(9) $$E\left(T\right)=\left(\overset{n}{\underset{j=1}{\sum }}j\right)\cdot \left(\overset{N}{\underset{j=1}{\sum }}z_{j}p_{j}\right)$$

To find ascending genes, we use *w_i _*= *i*, *i *= 1, ..., *N*; while for descending genes, we use *w_i _*= *N*-*i*+1, *i *= 1, ..., *N*. The Cuzick statistic above is obtained as a *z*-score, hence, we can determine a *p*-value for each gene and identify whether a gene is monotonic and the extent of its monotonicity (a stronger monotonic gene with a smaller *p*-value) by referring to the *z*-score table. In this study, we compare the performance of the Cuzick-test approach and our MFSelector approach (shown in the Results section).

### The modified M statistic test

We have also performed a comparison with the modified M statistic test [16]. The modified M statistic test (a distribution-based statistical test) is used to analyze dose-response studies in microarray experiments and investigate a trend in the response level expression with respect to doses in multi-class array data. In order to effectively find monotonic genes, the modified M statistic test adopts the means ordering strategy that considers the order restriction of mean expressions (responses) regarding the increasing or decreasing doses through *K *stages, respectively. The modified M statistic test and several other statistical tests are further integrated into an R package called IsoGene [18]. However, the modified M statistic test is less effective because the existence of outliers in a stage strongly influences the order of mean responses (as shown in the Results section).

### Functional annotation

After getting the list of monotonic genes, we can sort them by DE_total _or *p*-value in ascending order to identify those genes with stronger monotonic patterns, and decide the threshold for the gene list based on monotonicity. In addition, we further sort these monotonic genes with the same DE_total _values according to their associated SVDE. The resulting gene list is fed into the web service tool DAVID Bioinformatics Resources http://david.abcc.ncifcrf.gov/ to identify correspondingly enriched biological processes by cross-referencing to the gene ontology database http://www.geneontology.org/.

## Results

### Results on the ESCN data set by MFSelector

As mentioned in the Materials and Methods section, we use two microarray data sets, i.e., embryonic stem cell neurogenesis (ESCN) and embryonic stem cell vasculogenesis (ESCV), to demonstrate the effectiveness of our algorithm in identifying monotonic genes during different stages of stem cell differentiation. The results of application of MFSelector to the ESCV data set are exhibited in Materials S1 (in Additional file 1).

Figure S2 (in Additional file 3) reveals that principal component analysis (PCA) based on 9,285 probe sets (obtained using t-test, *q*<0.01) clearly distinguishes embryonic stem cells (ESCs) from post-natal neural stem cells (NSCs). It also shows that embryoid bodies (EB), primitive ectoderm cells (PEL) and neural rosette cells are differentiated along the neural lineage as evidenced by moving toward post-natal neural stem cells. Figure S2 (in Additional file 3) also shows that the addition of basic fibroblast growth factor (bFGF or FGF2) [19] does not make dramatic transcriptome changes on either day 10 primitive ectoderm cells (d10 and d10+) or day 17 neural rosette cells (d17 and d17+). So samples in d10 and d10+ are considered to belong to the same stage in this experiment. Similarly, samples in d17 and d17+ are considered to be in the same stage also.

**Figure 5:**
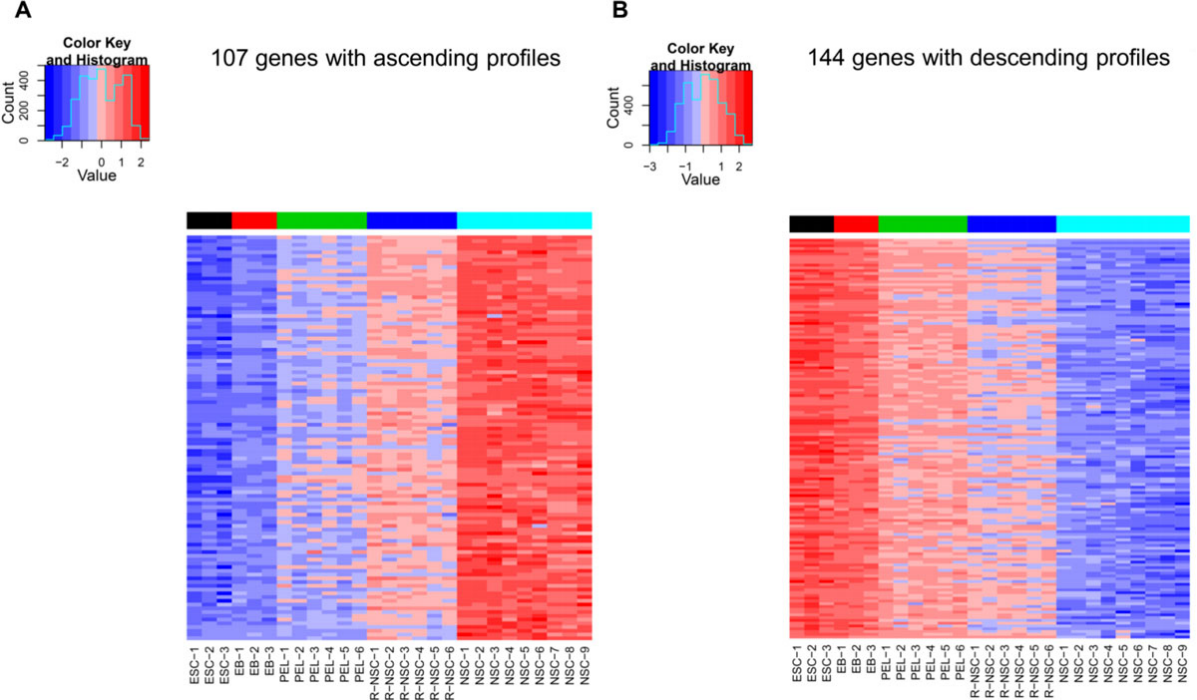
**Heatmaps of the two monotonic gene sets of the ESCN data set**. The x-axis represents samples in the five stages in stage order (the color label bar at the top represents the stage label, i.e., from black to aqua), the y-axis represents the set of genes in descending DE_total _order. The gene expression values change gradually from the blue band (low expression values) into the red band (high expression values) and vice versa. (A) 107 genes with ascending profiles and their range of DE_total _are from zero to three; (B) 144 genes with descending profiles and their range of DE_total _are from zero to three.

By applying our algorithm for the ESCN data set, we create gene lists for the ascending and descending nature respectively and sort these genes by the DE_total_. Using DE_total _<= 7 with the constraint *p*-value<1.0E-5 and with *N*-1 distinct discriminating lines (here *N *= 5), we get 857 ascending and 1,117 descending monotonic genes. The lists of these genes and correlated gene information are shown in Table S1 (in Additional file 4; available from: http://microarray.ym.edu.tw/tools/MFSelector/). Since there are too many monotonic genes whose *p*-value is less than 1.0E-5, we illustrate approximately one hundred top ranked ascending and descending genes from Table S1 (in Additional file 4) by using two heatmap visualizations in Figure 5. Figure 5(A) displays a heatmap of 107 ascending monotonic genes with DE_total _<= 3. Similarly, the gene expressions of the top 144 descending monotonic genes (DE_total _<= 3) are delineated in a heatmap in Figure 5(B). In Figure 5, the x-axis represents samples in the five stages in stage order (the color label bar at the top represents the stage label, i.e., from black to aqua), the y-axis represents the set of genes in descending DE_total _order. The gene expression values change gradually from the blue band (low expression values) into the red band (high expression values) in Figure 5(A), and vice versa in Figure 5(B), making the monotonicity easily discernible and obvious.

**Figure 6:**
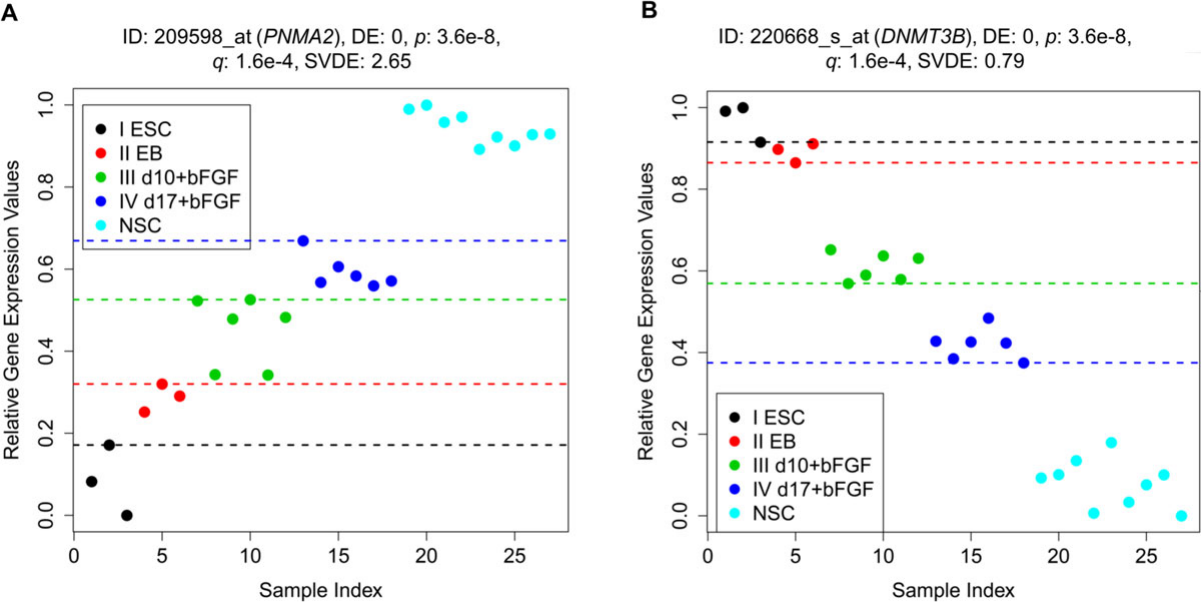
**Scatter plots of 209598_at (*PNMA2*) and 220668_s_at (*DNMT3B*) of the ESCN data set with ascending and descending profiles, respectively**. (A) This is one of the top three ascending genes with DE_total _= 0; (B) This is one of the top twelve descending genes with DE_total _= 0.

Biomarkers for a specific cell type exhibit high expression values when the stem cell is differentiated to this cell at the later stage of differentiation. This is a kind of stemness markers. Figure 6(A) depicts *PNMA2*, one of the top three ascending monotonic genes with DE_total _= 0 for the ESCN data set. For *PNMA2*, the samples from each stage are well distinguished from the samples from other stages sequentially because there is no discriminating error for every level of the process. A preliminary study indicates that *PNMA2 *(paraneoplastic antigen *MA2*, formerly known as *MA2*) is homologous to a recently cloned gene, *MA1*, which is a novel neuron- and testis-specific protein, and is recognized by the serum of patients with paraneoplastic neurological disorders [20]. Another two of the top three ascending monotonic genes (*PCDH9 *and *NPAS3*) also have correlation with neuronal receptor and neurogenesis [21,22]. Hence, the top three ascending monotonic genes identified by MFSelector from the ESCN data set are in fact reported to be related to neurogenesis. In addition, MFSelector has also identified *SOX1 *(DE_total _= 4), a famous marker for neural lineage cells [23].

Similarly, there are another kind of stemness markers which exhibit high expression at early stages of development and are monotonically descending during embryonic stem cell differentiation into the other cells/tissues (here, NSCs in the ESCN data set and vascular endothelial cells in the ESCV data set). Figure 6(B) displays *DNMT3B*, one of the top 12 descending genes with DE_total _= 0. Here, samples from all stages are also well separated by discriminating lines for each level. *DNMT3B *is associated with embryonic stem cell development [24]. Some of other top 12 descending genes are correlated with developmental pluripotency and are usually expressed in undifferentiated pluripotent stem cells (i.e., *EPCAM *and *DPPA4*) [25,26]. Thus, some of these top descending monotonic genes may play important roles in maintaining pluripotentiality. We shall further discuss some well-known embryonic stem cell related genes in Discussion section. Note that, we also identify some other previously unreported (or reported) genes of unknown function (or related to some biological function) with monotonic features.

In addition, we have checked one by one whether the top monotonic genes with DE_total _<= 3 (136 descending genes and 98 ascending genes with formal gene symbols) in the ESCN dataset are reported in the literature to be related to some stem cell characteristics, including stemness, differentiation, reprogramming, or induced pluripotent stem cell (iPS). Here we find that 54 (40%) monotonically *descending *genes related to some stem cell characteristics: stemness (44 genes), reprogramming (6 genes), and iPS (4 genes). Thirty-four (25%) genes are related to differentiation and some of them are related to embryogenesis. We could regard some of identified descending genes as stemness or embryo biomarkers. On the other hand, 30 (31%) monotonically *ascending *genes are found to be related to differentiation and most of them are related to neurogenesis (e.g., glia, nerve or other neural elements). We could also regard some of identified ascending genes as biomarkers of specific cell types in the differentiated cells. Please see Table S2 (in Additional file 5).

Furthermore, we have also checked one by one whether the top monotonic genes with DE_total _<= 3 in the ESCN dataset have a bi-stable profile with a significant drop or rise in expression level between two successive stages. We subjectively find that about 11 (11%) ascending genes and 17 (12.5%) descending genes are likely to have bi-stable switches. In the Table S2 (in Additional file 5), we have discussed some of those genes that are reported in the literature to be related to some stem cell characteristics.

**Figure 7:**
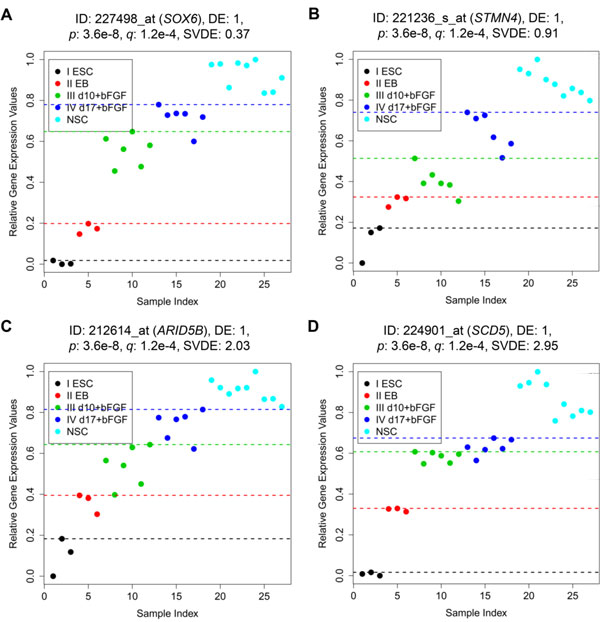
**Scatter plots of the four monotonic genes of the ESCN data set, whose DE_total _values all are equal to one, illustrate the sample variance for discriminating error (SVDE) by adding noise to each sample for 100 simulations**. (A) 227498_at (*SOX6*) with SVDE = 0.37 (1^st^); (B) 221236_s_at (*STMN4*) with SVDE = 0.91 (2^nd^); (C) 212614_at (*ARID5B*) with SVDE = 2.03 (3^rd^); (D) 224901_at (*SCD5*) with SVDE = 2.95 (4^th^).

To differentiate genes with the same DE_total_, we provide the SVDE value for each monotonic gene in Table S1 (in Additional file 4). Figure 7 shows the gene expression patterns of four ascending monotonic genes with the same DE_total_/*p*-value (DE_total _= 1). These four genes are distinguished by the SVDE values obtained by adding noise to the expression value and repeating the process 100 times. Figure 7(A) reveals that *SOX6*, with SVDE = 0.37, is the most monotonic gene compared to the other three genes in Figure 7, because its samples from different stages are well separated, except for a single discriminating error for Stage Four. Thus the DE_total _is not easily changed (i.e., a lower SVDE value) after adding noise to each sample. Figure 7(B) shows that *STMN4 *with SVDE = 0.91 is the second most monotonic gene, because the expression values of two samples, one from Stage Three and another from Stage Four, are very close. Therefore, the number of misclassified samples (DE_total_) for *STMN4 *is changed more frequently than *SOX6 *during the 100 experiments after adding noise to each sample. Visual inspection of the figures also suggests that *STMN4 *is the next monotonic gene in the list of four. Figure 7(C) indicates that *ARID5B *with SVDE = 2.03 is the third most monotonic gene. This is indeed the case because every two adjacent stages contain samples which have close expression values. Finally, Figure 7(D) displays *SCD5 *with SVDE = 2.95 the least monotonic of the four genes. A careful inspection of the figures also reveals the same and this is so because samples from Stage Three and Stage Four have expression values which are too close to avoid misclassification during the SVDE process.

### Comparison among three methods based on the ESCN data

Since the Cuzick-test can find many monotonic genes with the same rank, for preliminary comparison we have decided to use ascending genes with rank up to 20 as determined by the Cuzick-test as an example. For Cuzick-test, the number of genes selected with ranks 1~20 is 145. Then we use the top 145 monotonic ascending genes identified by each of the other methods, to find the number of common and unique identified genes. From Table S3 (in Additional file 6), we find that a large number of common genes (110 genes; 76%) are discovered by the Cuzick-test and MFSelector. On the other hand, a large number of unique genes are discovered by the modified M statistic test (only 50 (34%) common genes found with MFSelector and 54 (37%) common genes found with Cuzick-test). The detail results are shown in Table S3 and S4 (in Additional files 6 and 7) for ascending and descending cases, respectively. These findings suggest that the performances of the Cuzick-test and MFSelector are more similar than the performances of the modified M statistic test and MFSelector. However, there are some drawbacks in the Cuzick-test, which will be revealed when we further evaluate the three methods on the ESCN data set and the synthetic data sets.

#### Comparison between results by the Cuzick-test and MFSelector

For comparison with MFSelector, we have also applied the Cuzick-test to the ESCN data set and the ESCV data set, and determined the trends of the gene expression profiles. For illustration we have used only the ESCN data set. The results demonstrate several superior characteristics of MFSelector.

First, although the Cuzick-test can identify monotonic genes, the Cuzick-test results contain some non-monotonic genes (false positives) at or near the top of the gene lists. Figure S3 (in Additional file 8) shows four genes identified by the Cuzick-test as strong monotonic genes in terms of *z*-score/*p*-value because only samples from one stage (Stage Two) are out of the monotonic trend. Figure S3(A) and (B) (in Additional file 8) display two genes with only roughly ascending features as a result of the deviation from the trend in the expressions of samples from Stage Two. Figure S3(C) and (D) (in Additional file 8) display two other genes with only roughly descending features as a result of the deviation from the trend in the expressions of samples also from Stage Two. Note that, MFSelector discards most of this kind of genes with a partial monotonic trend (even though those genes have small DE_total _values) by examining whether we can determine their *N*-1 distinct discriminating lines.

Second, MFSelector uses SVDE to further determine different levels of monotonicity of genes with the same DE_total _values (*p*-values). Actually, many monotonic genes ranked by the Cuzick-test have the same *p*-value. For example, all of those genes with DE_total _= 0 always belong to the Cuzick-test top level. Figures S4(A) and (B) (in Additional file 9) show two genes with DE_total _= 0 that should have different levels of monotonicity based on their respective SVDE values. Similarly, Figs. S4(C) and (D) (in Additional file 9) also indicate different monotonicity under MFSelector SVDE analysis for two other genes which have the same DE_total _values (here DE_total _= 2) and both are ranked 10^th ^in the list generated by the Cuzick-test.

In addition, the Cusick-test may assign a low rank to a highly monotonic gene that contains some outlier samples which in fact do not adversely affect monotonicity. Figure S5(B) (in Additional file 10) shows that the gene expression value for *ZNF302 *is 164^th ^in the list generated by MFSelector, but is 105^th ^(in fact, its effective rank is 1279 because there are at least 1278 genes before the gene in the ranked list) in the list generated by the Cuzick-test because two samples from Stage One and Four, respectively, are significantly distant from its stage (they totally cross all samples of Stage Two and Three in expression value). This situation arises because this gene's ranking is significantly affected by the weight assigned to the anomalous expression value for these samples from Stage One and Four, causing the poor ranking for this gene under the Cuzick-test. Figure S5(A) (in Additional file 10) also shows the same characteristic influenced by outlier samples. On the other hand, the Cusick-test may also give a high rank to a gene which is insufficiently monotonic as a result of too much overlapping, where the lack of monotonicity is quite clear. For example, those monotonic genes in Figs. S6 and S7 (in Additional files 11 and 12) have too much overlapping among stages.

#### Comparison between results by the modified M statistic test and MFSelector

By applying the modified M statistic test, we create two gene lists, one for the ascending nature and the other for the descending nature respectively, and sort these genes by their corresponding M values. We illustrate only the top hundred ranked ascending and descending genes by using two heatmaps in Figs. S8 and S9 (in Additional files 13 and 14). Figure S8(A) (in Additional files 13) displays the heatmap of one hundred ascending monotonic genes produced by MFSelector and Fig. S8(B) (in Additional files 13) displays the same produced by the modified M statistic test. Similarly, the gene expressions of the top hundred descending monotonic genes chosen by MFSelector and the modified M statistic test are delineated in heatmaps in Figs. S9(A) and (B) (in Additional files 14), respectively. Note that, genes chosen from the top hundred are further clustered using hierarchical clustering with the average linkage strategy. It is evident from the yellow rectangular area on the heatmaps created from the gene lists (Figs. S8(B) and S9(B)), the genes found by the modified M selector method are not monotonic. This is a consequence of the existence of outliers in a stage (expressed by few different colors in a stage) satisfying the means ordering constraint. On the other hand, the genes found by MFSelector actually have monotonic trend (Figs. S8(A) and S9(A)).

### Results on the synthetic data

Nine pseudo genes are chosen randomly from the 9 types of monotonic genes as shown in Figure 1. We also use synthetic data sets to perform extensive comparisons, including quantitative and qualitative evaluations, between MFSelector, the Cuzick-test, and the modified M statistic test.

#### Comparison among three methods based on the synthetic data

In the ideal ranking, the top 60 pseudo genes should belong to the type 'Good (distinct/close)' (20 genes), 'Slightly' (20 genes), and 'Outliers (slight/severe)' (20 genes), and that is what MFSelector does. The results using the Cuzick-test are the approximately the same as that by MFSelector, but the modified M statistic test always selects about fifteen percent of genes in the type 'Partially ordered' which are worse cases and are out of monotonic trend (as shown in Additional files 15 and 16: Table S5 and S6). Nevertheless, the Cuzick-test and the modified M statistic test are seriously affected by the variation in the nature of outliers such as "slight" versus "severe" and the nature of partially ordered trends such as "far" versus "close". We think that both 'Outliers' type genes, slight or severe, should have similar monotonic trends. These outlier samples should not influence the extent of monotonic trends. Similarly, in the 'Partially ordered' type genes, no matter how significantly far the samples in one of the stages are from the monotonic trend in a gene expression profile, most of those genes should not influence the degree of monotonicity. In addition, as mentioned earlier, MFSelector discards most of this kind of genes with a partial monotonic trend (even though those genes have a small DE_total _values) by examining the number of distinct discriminating lines. Therefore, genes having slight/severe outliers or having partially ordered patterns with the level 'far' or 'close' should be given the similar significance/rank. For the synthetic data set, we find that the ranks of the pseudo genes *g46 *(with slight outlier characteristics) and *g53 *(with severe outlier characteristics) discovered by MFSelector are not different (similar DE_total _value) but their ranks are significantly different for the Cuzick-test and the modified M statistic test. The ranks of the pseudo genes *g102 *(with a close partially ordered stage) and *g111 *(with a far partially ordered stage) identified by MFSelector are also not different (same DE_total _value) but the ranks suggested by the Cuzick-test and the modified M statistic test are significantly different (as shown in Figure 1(D), (E), (H), and 1(I) and Additional file 15: Table S5).

## Discussion

### Biological relevance of other monotonic genes

Expressions of monotonic genes in different stages of cell lineage are closely related to the function of specific cell stage. Monotonicity of genes over stages is a good rationale to identify biomarkers responsible for differentiation of stem or precursor cells. For example, biomarkers for stemness exhibit high expression at early stages and those are responsible for maintaining proliferation and self-renewal activities. By contrast, biomarkers of specific cell types exhibit high expression in later stages after differentiation. And those genes also contribute to the specific phenotype and function of differentiated cells such as development of neural system which is mentioned earlier.

**Figure 8:**
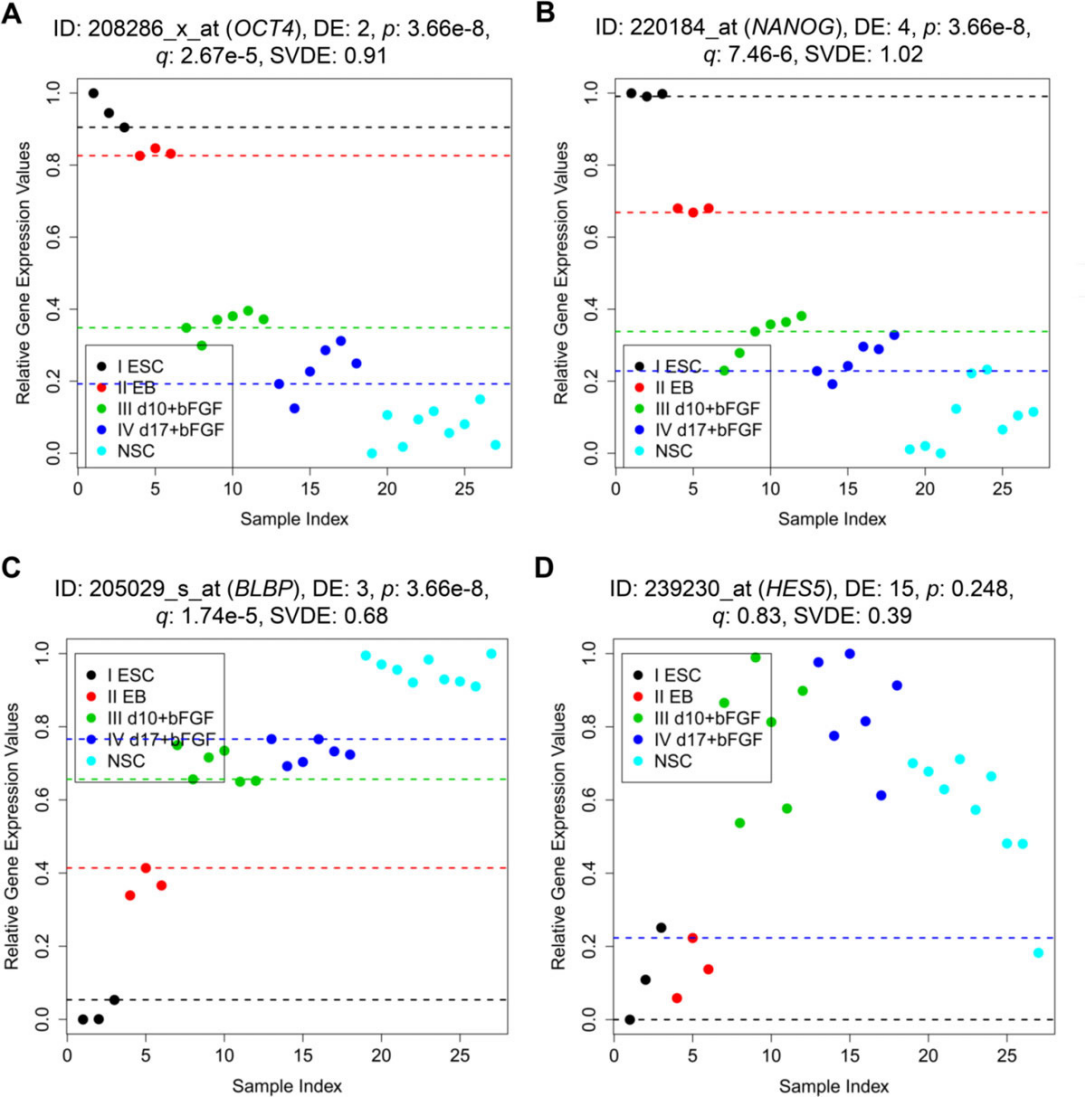
**Scatter plots of the four genes of the ESCN data set reported in the previous study **[1]. (A) 208286_x_at (*OCT4*); (B) 220184_at (*NANOG*); (C) 205029_s_at (*BLBP*); (D) 239230_at (*HES5*).

Among the top ascending and descending monotonic genes for the ESCN data set (shown in Additional file 4: Table S1), we have found many well-known and annotated marker genes proven and reported in previous studies. For example, *POU5F1 *(*OCT4*), *NANOG*, and *BLBP *(*FABP7*) (shown in Figures 8(A), (B) and 8(C), respectively) have been identified as important marker genes (with the same monotonic trends as observed in our study) in a microarray time series analysis (a neurogenesis experiment from day 0 to day 8) and further validated using RT-PCR [1]. *POU5F1 *(DE_total _= 2, number 39 in our list of descending monotonic genes) is a well-known transcription factor for maintaining and introducing stemness into embryonic stem cells [1]. *POU5F1 *is also important for pluripotency of embryonic stem cells [27]. *NANOG *(DE_total _= 4), is a well-known transcription factor critically involved with self-renewal of undifferentiated embryonic stem cells, and *BLBP *(*FABP7*; DE_total _= 3) is a neuroepithelial progenitor marker [1]. Furthermore, as reported earlier, *BLBP *is always expressed during development in radial glia by the activation of Notch receptors [28]. The expression of *BLBP *is usually induced in neural progenitor cells via Notch-1 activation [29]. Note that, in the previous study authors have also reported another important gene, *HES5 *with some ascending monotonic characteristics. This is a neuroepithelial progenitor marker [1] that mainly regulates brain development process [30]. It is interesting to observe that, while in the previous study, *HES5 *has been characterized as a monotonic ascending gene by their microarray data analysis, but in our study, Figure 8(D) clearly shows that *HES5 *expression for stages 1-4 is up-regulated and it is down-regulated for stage 5. Our finding is consistent with that of the previous study [1], which suggests that this gene is usually expressed in the rosette stage (about day 8) and stage 5 is beyond day 8. Consequently, in their RT-PCR experiment (until day 20), the same up- and down-regulated variance in expression patterns as shown in Figure 8(D) can be observed. While in our study, MFSelector does not define such genes with partial monotonic characteristics as good monotonic genes. For *HES5 *it has a very high DE_total _= 15. In future studies we may consider redefining DE values to include partial monotonicity.

Another research [31] has also reported a gene list comprised of known ESC-specific genes and some new candidates (such as *DNMT3B*, *LIN28A*, *BTF3 *and *ERH*) that can serve as markers for human embryonic stem cells and may also contribute to the stemness phenotype. Of particular interest in that study is the strong monotonic descending pattern of *DNMT3B *(DE_total _= 0) in our study during embryonic stem cell differentiation. *LIN28A *is without *N*-1 distinct discriminating lines because its expression is tightly grouping from Stage One to Stage Three. It has a small number of discriminating errors (DE_total _= 4) though. Additional transcription factors that were also highly expressed in human embryonic stem cells include *BTF3 *and *ERH*. These genes are marker genes for embryonic stem cell, which show high expressions only in human embryonic stem cells [31]. We depict the expression profile of these four genes in the ESCN data set in Fig. S10 (in Additional file 17). These scatter plots (as shown in Figs. S10(A), (B) and (C)) fit within the scenario for embryonic stem cell development explained by that study [31]. However, the expression profile of *ERH *over different stage is not clearly separated and is not monotonically descending during the neurogenesis process (as shown in Fig. S10(D)). We make a hypothesis that *ERH *does not clearly exhibit monotonic expression during neurogenesis even though it is an embryonic stem cell specific marker gene. This new biomarker needs to be surveyed and studied further both in wet- and dry- laboratory experiments.

### Biological processes involved in monotonic genes

The genes expressed in an early stage of stem cells or in undifferentiated cells (or precursor cells) of stem cells are often regarded as stem cell biomarkers, which are thought to maintain the pluripotency of embryonic stem cells. Based on the concept of pluripotency, cell growth or proliferation and the self-renewal activity are the most representative characteristics of stem cells which are known to be sustained by multiple genes in both stem cells and precursor cells [32]. Differentiation of stem cells or precursor cells is accompanied by the loss of pluripotency, which suggests that the expression of stemness related genes will decrease as well. This provides a rationale for why those genes should be included in the descending gene list generated by MFSelector.

Logically, the differentiation program of stem cells and precursor cells is not only driven by gene regulation but also manipulated by the circumstances of differentiation [33]. Combination of multiple stimulations in distinct circumstances provides the specific niche for differentiating into certain cell lineage, which is triggered by growth factors, transcription factors, and the specific cell surface receptors. Different growth factors or transcription factors are accompanied by different signals for the activation of specific functional genes, which are responsible for the generation of specific cell lineage. Therefore, we believe that those functional proteins related to the specialized cell type, structures, and functions will be involved in the ascending gene list generated by MFSelector.

To validate our inferences, we corroborate the biological processes for those monotonic genes identified from the two data sets by Gene Ontology analysis on the DAVID web tool http://david.abcc.ncifcrf.gov/. This helps us find distinct genes expressed in certain organogenesis with different processes.

For the ESCN data set, the top 857 ascending monotonic genes (DE_total _= 0~7) found by MFSelector are subjected to Gene Ontology analysis. Some of the biological processes those genes are involved in are neuron differentiation (41 genes, *p*-value = 8.1E-9), regulation of neurogenesis (23 genes, *p*-value = 3.5E-8), regulation of nervous system development (24 genes, *p*-value = 1.2E-7), regulation of neuron differentiation (19 genes, *p*-value = 4.6E-7), neuron development (31 genes, *p*-value = 1.2E-6), neuron projection development (24 genes, *p*-value = 1.7E-5), cell morphogenesis involved in neuron differentiation (21 genes, *p*-value = 2.5E-5), axonogenesis (20 genes, *p*-value = 2.6E-5), neuron projection morphogenesis (21 genes, *p*-value = 3.2E-5), and regulation of axonogenesis (11 genes, *p*-value = 1.7E-5). These biological processes very likely play important roles in differentiation of ESC neurogenesis and the development of the organs or the tissues of the nervous system. There are other ascending genes involved in neurotransmission, such as regulation of axon extension (7 genes, *p*-value = 3.5E-5), axon guidance (14 genes, *p*-value = 5.8E-5), transmission of nerve impulse (26 genes, *p*-value = 3.2E-4), and neuron migration (9 genes, *p*-value = 1.3E-3). These monotonic genes directly indicate their importance during the development of the nervous system and the process of neurogenesis.

Similarly, some of the top 1,117 descending monotonic genes (DE_total _= 0~7) found by MFSelector are involved in basic DNA/RNA biological processes, such as translation (42 genes, *p*-value = 4.3E-9), RNA processing (57 genes, *p*-value = 7.8E-9), ribosome biogenesis (23 genes, *p*-value = 2.4E-8), ribonucleoprotein complex biogenesis (28 genes, *p*-value = 3.8E-8), rRNA processing (19 genes, *p*-value = 1.3E-7), and ncRNA metabolic process (29 genes, *p*-value = 1.8E-6). According to the common understanding about the characteristics of undifferentiated or early stage stem cells, basic cellular processes are crucial for maintaining the capacity of self-renewal and proliferation. In addition, these monotonic genes may be responsible for the basic metabolisms and the stability of chromosome structures in cells. Proliferation of cells must undergo the process of cell cycle, and genes involved in the mechanism of cell cycle are able to imply the progress of proliferation. In conformity with this, we notice that genes for M phase (32 genes, *p*-value = 9.3E-5), DNA replication (22 genes, *p*-value = 1.4E-4), M phase of mitotic cell cycle (23 genes, *p*-value = 5.3E-4), mitotic cell cycle (32 genes, *p*-value = 7.3E-4), nuclear division (22 genes, *p*-value = 1.0E-3), mitosis (22 genes, *p*-value = 1.0E-3), cell cycle process (43 genes, *p*-value = 1.1E-3), and cell cycle phase (34 genes, *p*-value = 1.2E-3) are in the descending monotonic list. This evidence suggests that the stem cell features in the early stage exist during neurogenesis.

## Conclusions

We have developed a novel and robust scheme to identify genes with monotonic patterns in multi-stage as well as in time-series genomic data. Our method is based on a concept of total discriminating error practically without requiring any assumption. Using our proposed scheme we have been able to reveal genes with gradually increasing or decreasing expression patterns during stem cell differentiation. Some of these genes are known stemness genes (such as *POU5F1 *(*Oct4*)), while many other genes have not been linked to stem cell neurogenesis or vasculogenesis before. In the ESCN and ESCV data sets monotonic features could be found even from heterogeneous gene expression profiles. In addition, we have used monotonic markers discovered from one data set to analyze another data set and obtained very interesting results. An R package implementing our algorithm and data sets can be found at: http://microarray.ym.edu.tw/tools/MFSelector/.

## Competing interests

The authors declare that they have no competing interests.

## Authors' contributions

HWW conceived that such a study is valuable for time-series array data analysis. HJS, GCT, CTL, IFC and NRP designed the analysis approaches. HWW, TYC and SJC collected microarray data sets. HJS carried out the implementation of data analysis. HWW, TYC, HHL and WCC provided biological guidance during the analysis process. HWW, NRP, IFC and HJS wrote the manuscript. All authors read and approved the final manuscript.

## Supplementary Material

Additional file 1 **Materials S1**. Supporting online materials for the main article. Click here for file

Additional file 2 Figure S1. Illustration of adding noise to original expression values of 223038_s_at (*FAM60A*) of the ESCN data set. The DE_total _is four after running a simulation with added noise. Click here for file

Additional file 3 **Figure S2**. A three-dimensional scatter plot of the ESCV data set analyzed by principal component analysis. Click here for file

Additional file 4 **Table S1**. The lists of monotonic genes and correlated gene information of the ESCN and ESCV data sets. Click here for file

Additional file 5 Table S2. Details for the top monotonic genes (DE_total _<= 3) identified by MFSelector along with information from the literature in support of the facts that those genes are related to some stem cell characteristics, including stemness, differentiation, reprogramming or induced pluripotent stem cells. Click here for file

Additional file 6 **Table S3**. The lists of the number of common or unique genes from the monotonic ascending gene set (the top 145 genes with ascending profiles) of the ESCN data set identified by MFSelector, the Cuzick-test, and the modified M statistic test. Click here for file

Additional file 7 **Table S4**. The lists of the number of common or unique genes from the monotonic descending gene set (the top 163 genes with descending profiles) of the ESCN data set identified by MFSelector, the Cuzick-test, and the modified M statistic test. Click here for file

Additional file 8 Figure S3. Scatter plots of the four false positive monotonic genes identified by the Cuzick-test from the ESCN data set. (A) 200794_x_at (*DAZAP2*); (B) 229824_at (*SHC3*); (C) 206424_at (*CYP26A1*); (D) 209122_at (*PLIN2*). Click here for file

Additional file 9 Figure S4. Scatter plots of the four monotonic genes with the same DE_total _values but different SVDE values identified from the ESCN data set. (A) 1559280_a_at (*LOC100506013*); (B) 223000_s_at (*F11R*); (C) 230896_at (*BEND4*); (D) 202234_s_at (*SLC16A1*). Click here for file

Additional file 10 Figure S5. Scatter plots of the two monotonic genes for the ESCN data set ranked low by the Cuzick-test but high by MFSelector. (A) 202668_at (*EFNB2*); (B) 228393_s_at (*ZNF302*). Click here for file

Additional file 11 Figure S6. Scatter plots of the four ascending monotonic genes from the ESCN data set ranked high by the Cuzick-test but low by MFSelector. (A) 225717_at (*KIAA1715*); (B) 226907_at (*PPP1R14C*); (C) 229126_at (*TMEM19*); (D) 244040_at (*KCNN3*). Click here for file

Additional file 12 Figure S7. Scatter plots of the four descending monotonic genes from the ESCN data set ranked high by the Cuzick-test but low by MFSelector. (A) 204765_at (*ARHGEF5*); (B) 201930_at (*MCM6*); (C) 219786_at (*MTL5*); (D) 225359_at (*DNAJC19*). Click here for file

Additional file 13 **Figure S8**. Heatmaps of the monotonic ascending gene set (the top 100 genes with ascending profiles) of the ESCN data set identified by MFSelector and the modified M statistic test, respectively. (A) The top hundred monotonic ascending gene set chosen by MFSelector; (B) The top hundred ascending gene set chosen by the modified M statistic test. Click here for file

Additional file 14 **Figure S9**. Heatmaps of the monotonic descending gene set (the top 100 genes with descending profiles) of the ESCN data set identified by MFSelector and the modified M statistic test, respectively. (A) The top hundred monotonic descending gene set chosen by MFSelector; (B) The top hundred descending gene set chosen by the modified M statistic test. Click here for file

Additional file 15 **Table S5**. The lists of the ranks of monotonic genes for the synthetic data sets identified by MFSelector, the Cuzick-test, and the modified M statistic test. Click here for file

Additional file 16 **Table S6**. The list of the number of top genes identified by MFSelector, the Cuzick-test, and the modified M statistic test, in each type of monotonic genes of the synthetic data sets. Click here for file

Additional file 17 Figure S10. Scatter plots of the four genes of the ESCN data set reported in the previous study[31] (A) 220668_s_at (*DNMT3B*); (B) 219823_at (*LIN28A*); (C) 214800_x_at (*BTF3*); (D) 200043_at (*ERH*). Click here for file

## Declarations

This work is supported by Ministry of Science and Technology (MOST) (MOST103-2221-E-010-015) and National Yang-Ming University (a grant from Ministry of Education, Aim for the Top University Plan). The funders had no role in study design, data collection and analysis, decision to publish, or preparation of the manuscript.

This article has been published as part of *BMC Genomics *Volume 16 Supplement 2, 2015: Selected articles from the Thirteenth Asia Pacific Bioinformatics Conference (APBC 2015): Genomics. The full contents of the supplement are available online at http://www.biomedcentral.com/bmcgenomics/supplements/16/S2
