# Supplementary material for: Discovering monotonic stemness marker genes from time-series stem cell microarray data
Source: BMC Genomics. 2015 Jan 21;16(Suppl 2):S2. doi: 10.1186/1471-2164-16-S2-S2 (PMC4331716; doi:10.1186/1471-2164-16-S2-S2)
Supplement: Additional file 1 — Materials S1. Supporting online materials for the main article. [file 1471-2164-16-S2-S2-S1.pdf]

Supporting online materials for

Discovering monotonic stemness marker genes from time-series stem cell microarray data

Hsei-Wei Wang[1,2,3,4]#, Hsing-Jen Sun[1]#, Ting-Yu Chang[3]#, Hung-Hao Lo[5], Wei-Chung Cheng[6], George C. Tseng[7], Chin-Teng Lin[8,9], Shing-Jyh Chang[10], Nikhil Ranjan Pal[11§] and I-Fang Chung[1,2§]

[§]Correspondence: nikhil@isical.ac.in and ifchung@ym.edu.tw

[†] The software is available online http://microarray.ym.edu.tw/tools/MFSelector/

1. Microarray data collection and preprocessing
2. Results of ESCV
3. Biological relevance of other monotonic genes in the ESCV data set
4. Biological processes involved in monotonic genes in the ESCV data set
5. Supplemental Figures
6. Supplemental References

1. Microarray data collection and preprocessing

All data sets were downloaded from the NCBI GEO public archive, generated in Affymetrix Human Genome U133 Plus 2.0 platform with 54675 probe sets on chips, and the raw data (CEL file) normalized by RMA algorithm using the ‘affy’ package of the Bioconductor (http://www.bioconductor.org) [1] software suite in the R Project for statistical Computing (http://www.r-project.org). The default RMA settings are used for background correction, normalization and summarization of all expression values. The processed data matrices can be downloaded from the website: http://microarray.ym.edu.tw/tools/MFSelector/. In addition, the expression values of each gene are normalized in the range from zero to one across samples. This step preserves the richness in the original expression values for each gene among the samples, and helps us easily visualize the distribution of expression values for the significant genes. The PCA plots (generated by the statistical software, Partek® Genomics Suite™) are used for helping us understand the sample groupings.

1.1 Embryonic Stem Cell Neurogenesis data set (ESCN)

This data set contains 27 samples over five periods of human embryonic development: three embryonic stem cell (ESC) samples, three embryoid body (EB) samples, six primitive ectoderm cell (PEL) samples, six neural tube-like rosette cell samples, and nine post-natal neural stem cell (NSC) samples. This ESCN data set was downloaded from the NIH Neuroscience Microarray Consortium (GEO accession numbers are GSE9940 and GSE13307 [2]). In this data set, Zhang et al. (2008) first re-created in culture the developmental events of the first two to three weeks of human embryonic development during which embryonic stem (ES) cells were differentiated through the stages of embryoid bodies (EBs), primitive ectoderm cells (PEL), and neural tube-like rosette cells. The stage-specific events were then defined by Affymetrix Human Genome U133 Plus 2.0 array analysis along with the characteristic morphologic changes. Total RNAs were extracted from cells at the following neural specification developmental stages: ES cells (ESC, three samples), EBs grown in suspension (day 6, three samples), PEL stage (day 10, six samples), and neural rosettes (day 17, six samples). The post-natal neural stem cell (NSC, nine samples) array data are obtained from GSE13307 and our own home-made data [3].

1.2 Embryonic Stem Cell Vasculogenesis data set (ESCV)

In this data set, there are 13 samples over four periods of human embryonic stem cell differentiation into human mature (vascular) endothelial cells: three undifferentiated embryonic stem cell (ESC) samples, three mesodermal progenitor cell (MPC) samples, four embryoid body (EB) samples and three human mature vascular endothelial cell (VEC) samples. The ESCV data set is obtained by combining the following 2 differentiation data sets: GSE19735 [4] and GSE21668 [5]. The ESC samples are obtained from GSE21668. The three MPC samples (also from GSE21668) are extracted from the day 3.5 mesodermal progenitor (CD326neg CD56+) population. Mesoderm induction from human embryonic stem cells is initiated with combination of morphogens and growth factors including activin A, bone morphogenic protein 4, basic fibroblast growth factor and vascular endothelial growth factor. The mesodermal progenitor population is isolated by fluorescence-activated cell sorting (FACS) on day 3.5 of the culture based on the presence of CD56 expression and the absence of CD326 expression. The four EB samples (from GSE19735) are taken from two parts: one from human ESC EBs differentiated for 14 days in pro-angiogenic cytokines and the other from purified human ESC-derived durable endothelial cells isolated at day 14th of differentiation in the presence of TGFbeta inhibition.

2. Results of ESCV

The ESCV data set shows that vasculogenesis involves the differentiation of embryonic stem cells into mesodermal progenitor cells, which thereupon differentiate into embryoid bodies. The 18,046 probe sets (obtained using t-test, $q<0.01$) distinguishing embryonic stem cells (ESCs) from mature VECs show that mesodermal progenitor cells (MPC) are also differentiated along the vasculogenic lineage into embryoid bodies (EB), as verified by the development of these ESCs toward mature VECs (shown by PCA plot in Fig. S11).

Applying our algorithm to the ESCV data set, we obtain the 216 ascending and 563 descending monotonic genes using $DE_{total}=0$ with the constraint p-value<1.0E-5 and with N-1 distinct discriminating lines (here $N=4$). These genes are also included in Table S1 (in Additional file 4). Figure S12(A) displays the heatmap of the top 216 ascending (monotonic) genes with $DE_{total}=0$. Similarly, the gene expressions of the top 563 descending monotonic genes ($DE_{total}=0$) are shown as a heatmap in Fig. S12(B). These figures, like the ESCN data set, reveal the quality of the monotonic genes identified by our method.

One of the top 216 ascending (monotonic) genes for this data set is *JAK1* (Fig. S13(A)), which has been reported to be significantly involved in the interferon-alpha/-beta and -gamma signal transduction pathways [6]. Some of the other top 216 ascending monotonic genes, such as *PDE8A* [7], *ACTR2* [8], *TPM4* [9-11], *ANXA2* [12], *PON2* [13, 14] and *ADAM9* [15, 16] have been reported to be expressed in vascular endothelial cells, vascular smooth muscle proliferation, neovascularization or have effect on heart/cardiovascular tissue.

On the other hand, one of the top 563 monotonically descending genes with $DE_{total}=0$ during ESC vasculogenesis is *DDR1* (Fig. S13(B)). *DDR1* is one of the cell adhesion molecules and it plays a key role in the cell-cell interactions [17]. In addition, *FOXC2* transcription factor (forkhead box C2, mesenchyme forkhead 1; $DE_{total}=0$) is crucial for the induction of in vivo endothelial gene expression during endothelial cell differentiation from the primitive mesodermal cells [18]. And *FOXC2* is also a crucial regulator involved in lymphangiogenesis [19]. Some of the other top 563 descending monotonic genes, such as *RBM25* [20], *CTBP2* [21], *PABPN1* [22], *RB1CC1* [23], *FAN1* [24], are shown to be related to key biological processes such as alternative pre-mRNA splicing/transcript, cell death, DNA replication or DNA repair. The genes *TACC3* [25], HSPA9 [26], *TCOF1* [27] and *CHAF1A* [28], which are also in the list of top 563 descending genes, have correlation with cell growth/proliferation, cell differentiation or embryonic development.

For this data set also we demonstrate the effectiveness of SVDE on four genes with $DE_{total}=0$. These genes are depicted in Fig. S14. These four genes are distinguished based on the SVDE values. Inspection of Fig. S14(A) suggests that *CAPNS1* has the highest degree of monotonicity compared to the other three genes in Fig. S14, and this is indeed conformed by the SVDE values. Figure S14(B) shows that *NNMT* with SVDE=0.96 is the second most monotonic gene. This agrees with our expectation from visual inspection. *NNMT* is less monotonic than *CAPNS1* because the expression values of two samples, the third sample from Stage Three and the first sample from Stage Four, are very close. Based on the SVDE values we find that *GUK1* (Fig. S14(C)) is the third most monotonic one while *TMEM45B* (Fig. S14(D)) is least monotonic of the four. This conclusion based on SVDE agrees with our visual assessment of the figures.

3. Biological relevance of other monotonic genes in the ESCV data set

Some genes with significantly descending characteristics are the subject of interest because of their rules and/or regulation during stem cell differentiation and vasculogenesis. For example, *CBX3* ($DE_{total}=1$) is a monotonic gene with a strong descending attribute in the ESCV data set. As described earlier, *CBX3* plays a significant role in

DNA repair and cell lineage differentiation. *CBX3* directly promotes stem cell differentiation into smooth muscle cells, which participates in the development of the vascular system in the embryonic stage [29]. Furthermore, two descending genes, *SMAD4* (DE_{total}=1) and *MDM4* (DE_{total}=1) are responsible for stemness in the early stage of differentiation. *SMAD4* is considered a factor for stabilizing the state of undifferentiated embryonic stem cells and the reduction of *SMAD4* affects the stability of stem cells and drives the differentiation of stem cells into a particular cell lineage [30]. *SMAD4* also regulates self-renewal and pluripotency through a complicated mechanism via the TGF-β signaling pathway. This signaling maintains the stemness of embryonic stem cells in an early stage with an undifferentiated state. Although the role of *MDM4* (another monotonic gene identified by MFSelector) in embryonic stem cells remains unclear, *MDM4* is known as a negative regulator of p53 protein [31]. *MDM4* can directly interact with the transactivation domain of p54 for inhibiting the function of p53. Genetic defects in *MDM4* leads to embryonic lethality, which is mediated by abolishing the regulatory mechanism to p53 [32]. Recent studies also reported that *MDM4* directly down-regulates the transcriptional activity of p53 during the process of embryonic stem cell differentiation [33]. Through the regulation of p53, *MDM4* modulates cell survival during the differentiation of embryonic stem cells. Noticeably, *NCL* (DE_{total}=1) also shows down-regulation through the progress of vasculogenesis. *NCL* is a transcription activator of VEGF promoter [34]. As described above, *VEGF* is a well-known growth factor for inducing and stimulating vasculogenesis. Expression of *NCL* in early stage of embryonic stem cell is a crucial switch for activating *VEGF* expression. Whenever the level of *VEGF* is elevated, which directly promotes cell differentiation and processing vasculogenesis, *NCL* will no longer express with high level and will show a descending pattern.

On the other hand, some of the ascending genes, found by MFSelector, have also been proven to have functions in vascular development. For example, *SOX17* (DE_{total}=1), a transcription factor, is a differentially expressed temporal specification of blood progenitors from mouse embryonic stem cells and induced pluripotent stem cells (iPS cells) [35]. *PECAM1* (platelet endothelial cell adhesion molecule; DE_{total}=1), also known as *CD31*, has been widely reported to be involved in vascular endothelial cell integrity/formation [36-38]. *PGF* (DE_{total}=2), also named *PIGF*, is a member of *VEGF* family. *PGF* acts as an agonist for *VEGFR1*, which is ubiquitously expressed in the endothelial cell lineage. Several studies have reported significant role of *PGF* in vessel formation or tumor vasculogenesis [39, 40]. Another important gene found by MFSelector is *RhoB* (DE_{total}=1), which plays a unique role in several cellular processes, such as vesicle trafficking, Akt control, and cell survival. Through the regulation of *Akt*, *RhoB* maintains the stability and survival of endothelium during the process of vascular development. A recent study has demonstrated that the role of *RhoB* in the regulation of endothelial cell survival during vasculogenesis is in a stage-specific manner. The function of *RhoB* has also been evaluated through the *RhoB* knockout mice, which results in defects during the development of the vascular system. Moreover, another characteristic of *RhoB* is to maintain the angiogenesis progress through suppression of the anti-angiogenic agents. The stage-specific functions of *RhoB* in vasculogenesis make it a meaningful marker for the progression of vasculogenesis [41]. Epidermal growth factors (EGFs) are the major contributors to the growth and survival of endothelial cells, including embryonic endothelial progenitor cells. Another ascending gene, *NRG1* (DE_{total}=1), is a ligand of *EGF* family and it directly mediates the activity of endothelial progenitor cells and promotes angiogenesis [42]. In addition to EGF family, another growth factor *IGF-1* (DE_{total}=1) also has an ascending profile. As a paracrine and autocrine, *IGF-1* and *IGF-II* modulate the proliferation and differentiation during the process of embryogenesis [43]. *IGF-1*/*IGF-I* receptor systems are involved in vasculogenesis during the development of lung and other organogenesis. Through maintenance of the endothelial cell population as a survival factor, *IGF-1* promotes the development of the vascular system during the embryonic stage of rats. Higher expression of *IGF-1*

plays a crucial role in the process of vasculogenesis [44]. Notably, *TEK* (*TIE-2*; DE_{total}=2) is a receptor tyrosine kinase that is known to function as a molecule of vascular endothelial cells. *TEK* is found to be expressed specifically in the endothelial lineage and is required for the normal development of vascular structures during embryogenesis [45, 46]. In addition, *TEK* is also uniformly expressed in the endothelial lining of the vasculature [47]. Hence, we infer that *TEK* is usually expressed in endothelial cells of blood vessels during embryonic development.

4. Biological processes involved in monotonic genes in the ESCV data set

For the ESCV dataset, when the top 216 ascending monotonic genes (DE_{total}=0) found by MFSelector are subjected to GO analysis, few genes involved in ESCV-related processes, such as blood vessel morphogenesis (6 genes, *p*-value=0.035), blood vessel development (6 genes, *p*-value=0.06), vasculature development (6 genes, *p*-value=0.065), and striated muscle tissue development (5 genes, *p*-value=0.019) are found to be induced during ESC endothelial differentiation. These ascending genes directly guide the process of vasculogenesis and develop the basic structure of the vascular system. In addition to the functional genes related to vasculogenesis, other signal transducers such as VEGF or PGH are also involved during vascular development. As a ligand for VEGFR, VEGF transduces a significant signal for progression of angiogenesis. Hence, the genes related to cellular signaling transduction may sustain the function and activity of endothelial cells for angiogenesis or vasculogenesis. GO analysis reveals several genes with ascending profile during vasculogenesis for the signaling transduction system, such as cellular protein localization (11 genes, *p*-value=0.0279), cellular macromolecule localization (11 genes, *p*-value=0.0294), intracellular transport (14 genes, *p*-value=0.004), intracellular protein transport (10 genes, *p*-value=0.004), protein localization (16 genes, *p*-value=0.008), RAS protein signal transduction (5 genes, *p*-value=0.012) and RHO protein signal transduction (3 genes, *p*-value=0.041).

On the other hand, some of the top 563 descending monotonic genes with DE_{total}=0 are involved in DNA/RNA or other basic biological processes, such as DNA metabolic process (37 genes, *p*-value=2.9E-8), DNA replication (21 genes, *p*-value=9.1E-8), cell cycle (41 genes, *p*-value=0.000092), and chromosome organization (30 genes, *p*-value=0.000021). These genes with basic functions are responsible for the proliferation and replication of stem cells. Without any lineage differentiation and further stimulation, stem cells in an early stage do not possess specific functions and characteristics. Maintenance of high proliferation rate and chromosome stability is the most significant function in stem cells or precursor cells. Therefore, genes related to proliferative and self-renewal activities for maintaining the pluripotency are supposed to play important roles in undifferentiated cells, especially in stem cells. On the other hand, stimulation from circumstances may cause damage to genome structures. Hence we can find those genes with response to DNA damage stimulus (24 genes, *p*-value=9.62E-5) and DNA repair (20 genes, *p*-value=1.37E-4) as expressed in an early stage for stabilizing the genome structure.

Supplemental Figures

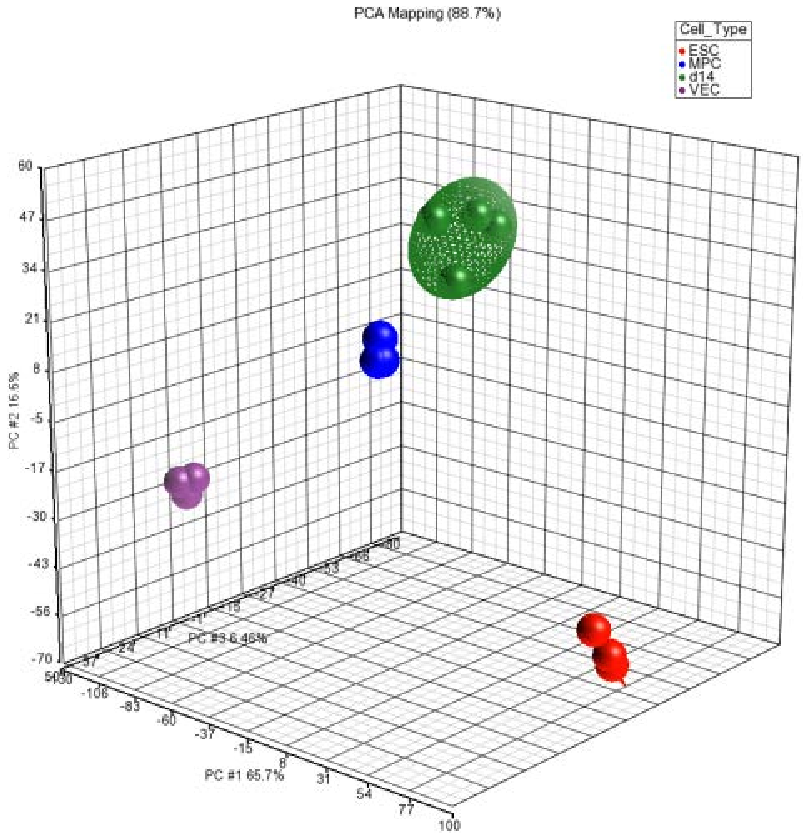

Fig. S11. A three-dimensional scatter plot of the ESCV data set analyzed by principal component analysis.

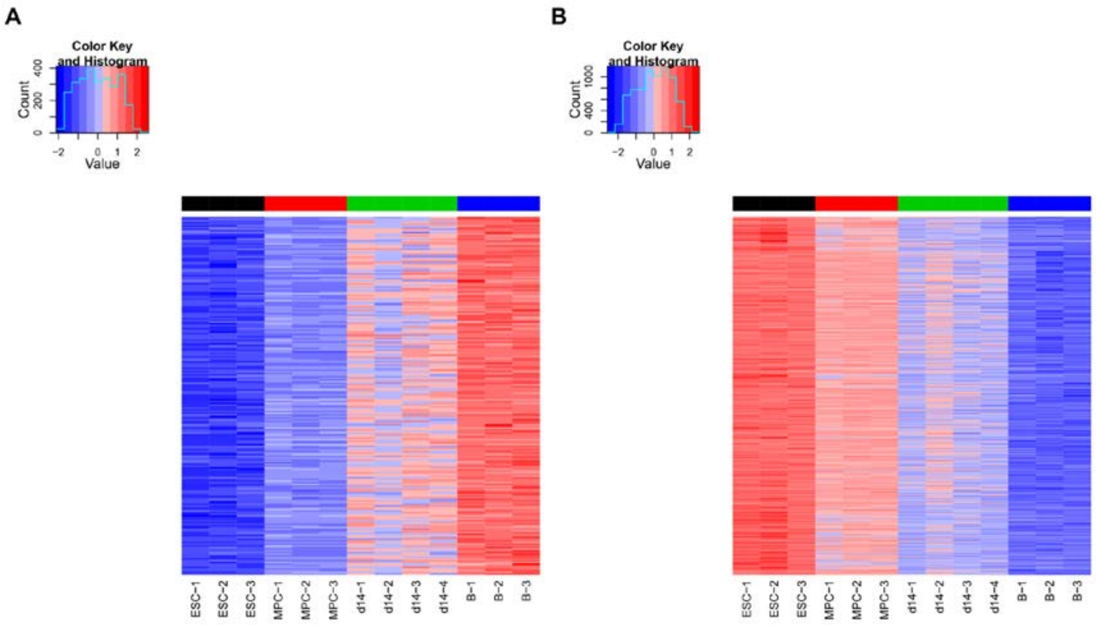

Figure S12. Heatmaps of the two monotonic gene sets of the ESCV data set. The gene expression values change gradually from the blue band (low expression values) into the red band (high expression values) and vice versa. (A) 216 monotonic genes with ascending profiles and their DEtotal values are zero; (B) 563 monotonic genes with descending profiles and their DEtotal values are zero.

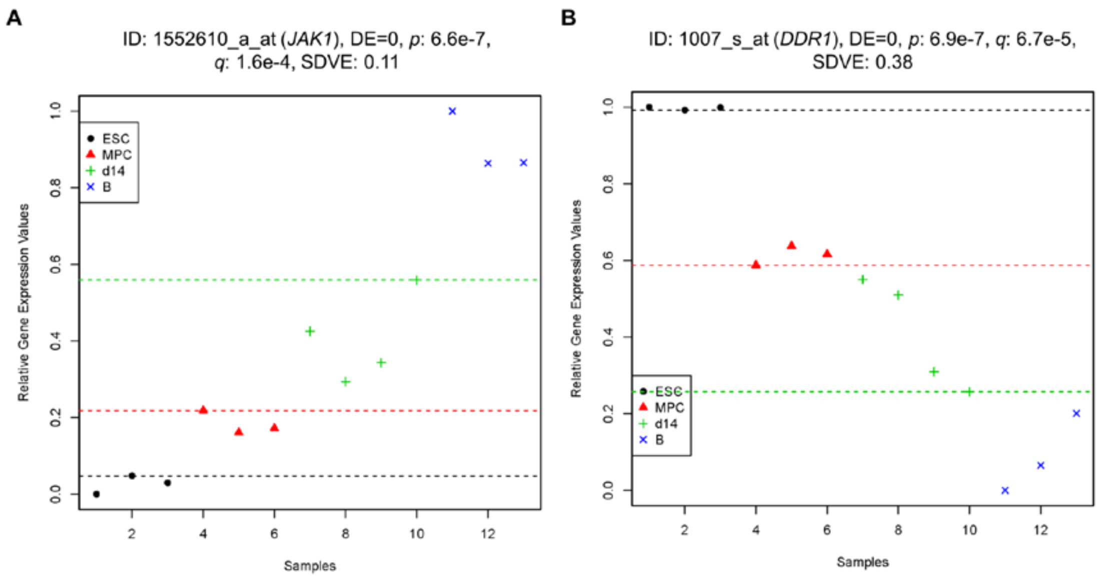

Fig. S13. Scatter plots of 1552610_a_at (*JAK1*) and 1007_s_at (*DDR1*) of the ESCV data set with ascending profile and descending profile respectively. (A) This is one of the top 216 monotonically ascending genes with DE_{total}=0; (B) This is one of the top 563 monotonically descending genes with DE_{total}=0.

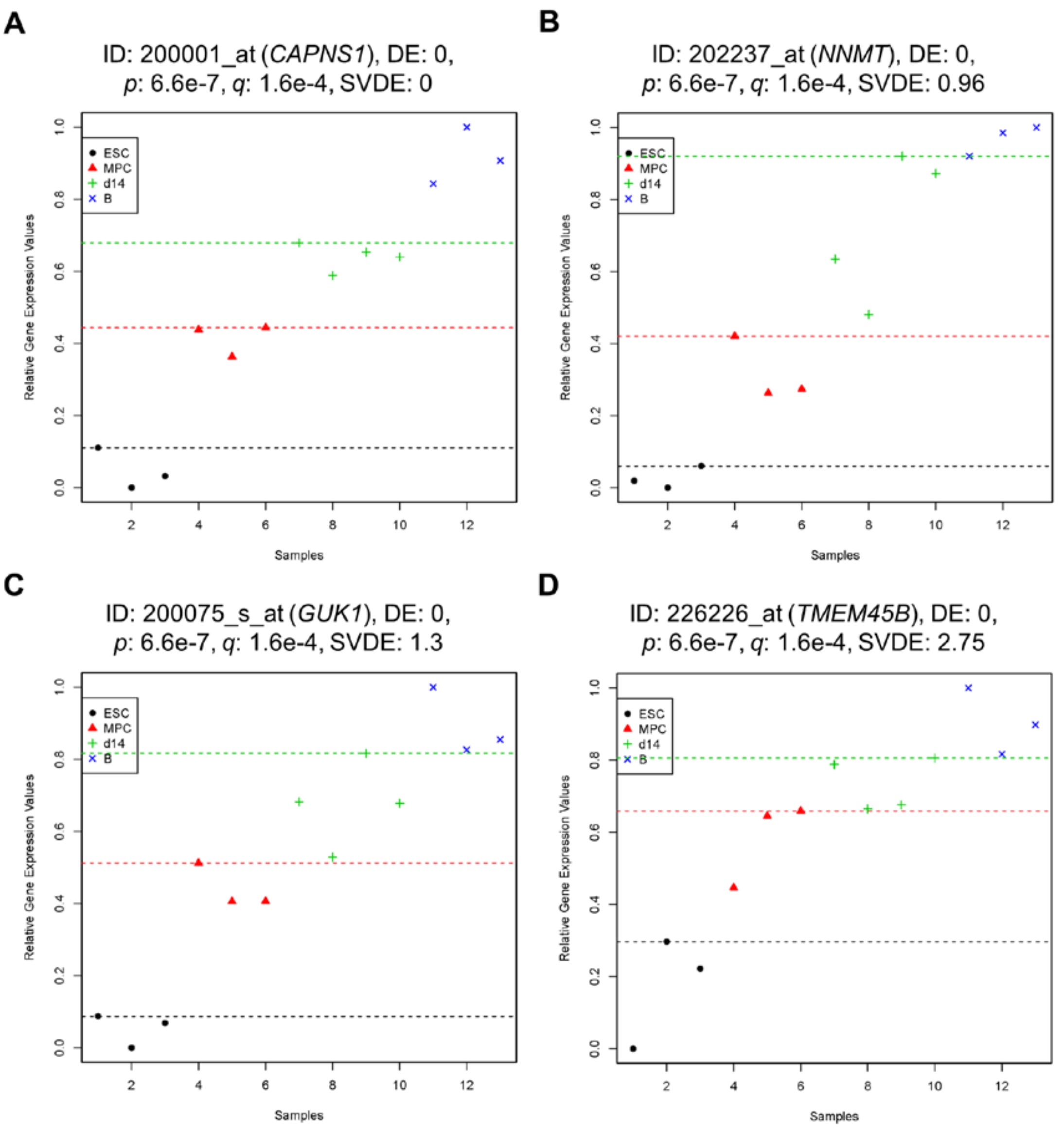

Fig. S14. Scatter plots of the four ascending monotonic genes of the ESCV data set, whose DE_{total} values all are equal to one, illustrate the sample variance for discriminating error by adding noise to each sample for 100 simulations. (A) 200001_at (*CAPNS1*) with SVDE=0 (1st); (B) 202237_at (*NNMT*) with SVDE=0.96 (2nd); (C) 200075_s_at (*GUK1*) with SVDE=1.3 (3rd); (D) 226226_at (*TMEM45B*) with SVDE=2.75 (4th).

Supplemental References

1. Gentleman RC, Carey VJ, Bates DM, Bolstad B, Dettling M, Dudoit S, Ellis B, Gautier L, Ge Y, Gentry J *et al*: **Bioconductor: open software development for computational biology and bioinformatics**. *Genome biology* 2004, **5**(10):R80.
2. Kim HJ, McMillan E, Han F, Svendsen CN: **Regionally specified human neural progenitor cells derived from the mesencephalon and forebrain undergo increased neurogenesis following overexpression of ASCL1**. *Stem Cells* 2009, **27**(2):390-398.
3. Huang TS, Hsieh JY, Wu YH, Jen CH, Tsuang YH, Chiou SH, Partanen J, Anderson H, Jaatinen T, Yu YH *et al*: **Functional network reconstruction reveals somatic stemness genetic maps and dedifferentiation-like transcriptome reprogramming induced by GATA2**. *Stem Cells* 2008, **26**(5):1186-1201.
4. James D, Nam HS, Seandel M, Nolan D, Janovitz T, Tomishima M, Studer L, Lee G, Lyden D, Benezra R *et al*: **Expansion and maintenance of human embryonic stem cell-derived endothelial cells by TGFbeta inhibition is Id1 dependent**. *Nature biotechnology* 2010, **28**(2):161-166.
5. Evseenko D, Zhu Y, Schenke-Layland K, Kuo J, Latour B, Ge S, Scholes J, Dravid G, Li X, MacLellan WR *et al*: **Mapping the first stages of mesoderm commitment during differentiation of human embryonic stem cells**. *Proc Natl Acad Sci U S A* 2010, **107**(31):13742-13747.
6. Gadina M, Hilton D, Johnston JA, Morinobu A, Lighvani A, Zhou YJ, Visconti R, O'Shea JJ: **Signaling by type I and II cytokine receptors: ten years after**. *Curr Opin Immunol* 2001, **13**(3):363-373.
7. Hetman JM, Soderling SH, Glavas NA, Beavo JA: **Cloning and characterization of PDE7B, a cAMP-specific phosphodiesterase**. *Proc Natl Acad Sci U S A* 2000, **97**(1):472-476.
8. McCarthy SA, Bicknell R: **Activin-A binds to a heterotrimeric receptor complex on the vascular endothelial cell surface. Evidence for a type 3 activin receptor**. *J Biol Chem* 1994, **269**(6):3909-3912.
9. Abouhamed M, Reichenberg S, Robenek H, Plenz G: **Tropomyosin 4 expression is enhanced in dedifferentiating smooth muscle cells in vitro and during atherogenesis**. *European journal of cell biology* 2003, **82**(9):473-482.
10. Gallant C, Appel S, Graceffa P, Leavis P, Lin JJ, Gunning PW, Schevzov G, Chaponnier C, DeGnore J, Lehman W *et al*: **Tropomyosin variants describe distinct functional subcellular domains in differentiated vascular smooth muscle cells**. *American journal of physiology Cell physiology* 2011, **300**(6):C1356-1365.
11. Zhao L, Zhao X, Tian T, Lu Q, Skrbo-Larssen N, Wu D, Kuang Z, Zheng X, Han Y, Yang S *et al*: **Heart-specific isoform of tropomyosin4 is essential for heartbeat in zebrafish embryos**. *Cardiovascular research* 2008, **80**(2):200-208.
12. Zhao SH, Pan DY, Zhang Y, Wu JH, Liu X, Xu Y: **Annexin A2 promotes choroidal neovascularization by increasing vascular endothelial growth factor expression in a rat model of argon laser coagulation-induced choroidal neovascularization**. *Chin Med J (Engl)* 2010, **123**(6):713-721.
13. Fuhrman B, Gantman A, Khateeb J, Volkova N, Horke S, Kiyan J, Dumler I, Aviram M: **Urokinase activates macrophage PON2 gene transcription via the PI3K/ROS/MEK/SREBP-2 signalling cascade mediated by the PDGFR-beta**. *Cardiovascular research* 2009, **84**(1):145-154.
14. Horke S, Witte I, Wilgenbus P, Kruger M, Strand D, Forstermann U: **Paraoxonase-2 reduces oxidative stress in vascular cells and decreases endoplasmic reticulum stress-induced caspase activation**. *Circulation* 2007, **115**(15):2055-2064.
15. Guaiquil V, Swendeman S, Yoshida T, Chavala S, Campochiaro PA, Blobel CP: **ADAM9 is involved in pathological**

retinal neovascularization. *Molecular and cellular biology* 2009, **29**(10):2694-2703.

16. Oksala N, Levula M, Airla N, Pelto-Huikko M, Ortiz RM, Jarvinen O, Salenius JP, Ozsait B, Komurcu-Bayrak E, Erginel-Unaltuna N *et al*: **ADAM-9, ADAM-15, and ADAM-17 are upregulated in macrophages in advanced human atherosclerotic plaques in aorta and carotid and femoral arteries--Tampere vascular study**. *Annals of medicine* 2009, **41**(4):279-290.
17. Heinzelmann-Schwarz VA, Gardiner-Garden M, Henshall SM, Scurry J, Scolyer RA, Davies MJ, Heinzelmann M, Kalish LH, Bali A, Kench JG *et al*: **Overexpression of the cell adhesion molecules DDR1, Claudin 3, and Ep-CAM in metaplastic ovarian epithelium and ovarian cancer**. *Clinical cancer research : an official journal of the American Association for Cancer Research* 2004, **10**(13):4427-4436.
18. De Val S, Chi NC, Meadows SM, Minovitsky S, Anderson JP, Harris IS, Ehlers ML, Agarwal P, Visel A, Xu SM *et al*: **Combinatorial regulation of endothelial gene expression by ets and forkhead transcription factors**. *Cell* 2008, **135**(6):1053-1064.
19. Wu X, Liu NF: **FOXC2 transcription factor: a novel regulator of lymphangiogenesis**. *Lymphology* 2011, **44**(1):35-41.
20. Zhou A, Ou AC, Cho A, Benz EJ, Jr., Huang SC: **Novel splicing factor RBM25 modulates Bcl-x pre-mRNA 5' splice site selection**. *Molecular and cellular biology* 2008, **28**(19):5924-5936.
21. Chinnadurai G: **CtBP, an unconventional transcriptional corepressor in development and oncogenesis**. *Molecular cell* 2002, **9**(2):213-224.
22. Calado A, Kutay U, Kuhn U, Wahle E, Carmo-Fonseca M: **Deciphering the cellular pathway for transport of poly(A)-binding protein II**. *Rna* 2000, **6**(2):245-256.
23. Chano T, Ikegawa S, Kontani K, Okabe H, Baldini N, Saeki Y: **Identification of RB1CC1, a novel human gene that can induce RB1 in various human cells**. *Oncogene* 2002, **21**(8):1295-1298.
24. MacKay C, Declais AC, Lundin C, Agostinho A, Deans AJ, MacArtney TJ, Hofmann K, Gartner A, West SC, Helleday T *et al*: **Identification of KIAA1018/FAN1, a DNA repair nuclease recruited to DNA damage by monoubiquitinated FANCD2**. *Cell* 2010, **142**(1):65-76.
25. Sadek CM, Pelto-Huikko M, Tujague M, Steffensen KR, Wennerholm M, Gustafsson JA: **TACC3 expression is tightly regulated during early differentiation**. *Gene expression patterns : GEP* 2003, **3**(2):203-211.
26. Wadhwa R, Yaguchi T, Hasan MK, Taira K, Kaul SC: **Mortalin-MPD (mevalonate pyrophosphate decarboxylase) interactions and their role in control of cellular proliferation**. *Biochemical and biophysical research communications* 2003, **302**(4):735-742.
27. Poswillo D: **The pathogenesis of the Treacher Collins syndrome (mandibulofacial dysostosis)**. *The British journal of oral surgery* 1975, **13**(1):1-26.
28. Polo SE, Theocharis SE, Klijanienko J, Savignoni A, Asselain B, Vielh P, Almouzni G: **Chromatin assembly factor-1, a marker of clinical value to distinguish quiescent from proliferating cells**. *Cancer Res* 2004, **64**(7):2371-2381.
29. Xiao Q, Wang G, Yin X, Luo Z, Margariti A, Zeng L, Mayr M, Ye S, Xu Q: **Chromobox protein homolog 3 is essential for stem cell differentiation to smooth muscles in vitro and in embryonic arteriogenesis**. *Arteriosclerosis, thrombosis, and vascular biology* 2011, **31**(8):1842-1852.
30. Avery S, Zafarana G, Gokhale PJ, Andrews PW: **The role of SMAD4 in human embryonic stem cell self-renewal and stem cell fate**. *Stem Cells* 2010, **28**(5):863-873.
31. Shvarts A, Steegenga WT, Riteco N, van Laar T, Dekker P, Bazuine M, van Ham RC, van der Houven van Oordt W, Hateboer G, van der Eb AJ *et al*: **MDMX: a novel p53-binding protein with some functional properties of MDM2**. *EMBO J* 1996, **15**(19):5349-5357.

32. Parant J, Chavez-Reyes A, Little NA, Yan W, Reinke V, Jochemsen AG, Lozano G: **Rescue of embryonic lethality in Mdm4-null mice by loss of Trp53 suggests a nonoverlapping pathway with MDM2 to regulate p53**. *Nature genetics* 2001, **29**(1):92-95.

33. Menendez S, Goh AM, Camus S, Ng KW, Kua N, Badal V, Lane DP: **MDM4 downregulates p53 transcriptional activity and response to stress during differentiation**. *Cell cycle* 2011, **10**(7):1100-1108.

34. Uribe DJ, Guo K, Shin YJ, Sun D: **Heterogeneous nuclear ribonucleoprotein K and nucleolin as transcriptional activators of the vascular endothelial growth factor promoter through interaction with secondary DNA structures**. *Biochemistry* 2011, **50**(18):3796-3806.

35. Irion S, Clarke RL, Luche H, Kim I, Morrison SJ, Fehling HJ, Keller GM: **Temporal specification of blood progenitors from mouse embryonic stem cells and induced pluripotent stem cells**. *Development* 2010, **137**(17):2829-2839.

36. Privratsky JR, Paddock CM, Florey O, Newman DK, Muller WA, Newman PJ: **Relative contribution of PECAM-1 adhesion and signaling to the maintenance of vascular integrity**. *Journal of cell science* 2011, **124**(Pt 9):1477-1485.

37. Ross EA, Freeman S, Zhao Y, Dhanjal TS, Ross EJ, Lax S, Ahmed Z, Hou TZ, Kalia N, Egginton S *et al*: **A novel role for PECAM-1 (CD31) in regulating haematopoietic progenitor cell compartmentalization between the peripheral blood and bone marrow**. *PLoS One* 2008, **3**(6):e2338.

38. Woodfin A, Voisin MB, Nourshargh S: **PECAM-1: a multi-functional molecule in inflammation and vascular biology**. *Arteriosclerosis, thrombosis, and vascular biology* 2007, **27**(12):2514-2523.

39. Burton GJ, Charnock-Jones DS, Jauniaux E: **Regulation of vascular growth and function in the human placenta**. *Reproduction* 2009, **138**(6):895-902.

40. Li B, Sharpe EE, Maupin AB, Teleron AA, Pyle AL, Carmeliet P, Young PP: **VEGF and PlGF promote adult vasculogenesis by enhancing EPC recruitment and vessel formation at the site of tumor neovascularization**. *FASEB journal : official publication of the Federation of American Societies for Experimental Biology* 2006, **20**(9):1495-1497.

41. Adini I, Rabinovitz I, Sun JF, Prendergast GC, Benjamin LE: **RhoB controls Akt trafficking and stage-specific survival of endothelial cells during vascular development**. *Genes & development* 2003, **17**(21):2721-2732.

42. Safa RN, Peng XY, Pentassuglia L, Lim CC, Lamparter M, Silverstein C, Walker J, Chen B, Geisberg C, Hatzopoulos AK *et al*: **Neuregulin-1beta regulation of embryonic endothelial progenitor cell survival**. *American journal of physiology Heart and circulatory physiology* 2011, **300**(4):H1311-1319.

43. D'Ercole AJ: **Somatomedins/insulin-like growth factors and fetal growth**. *Journal of developmental physiology* 1987, **9**(6):481-495.

44. Han RN, Post M, Tanswell AK, Lye SJ: **Insulin-like growth factor-I receptor-mediated vasculogenesis/angiogenesis in human lung development**. *American journal of respiratory cell and molecular biology* 2003, **28**(2):159-169.

45. Sato A, Iwama A, Takakura N, Nishio H, Yancopoulos GD, Suda T: **Characterization of TEK receptor tyrosine kinase and its ligands, Angiopoietins, in human hematopoietic progenitor cells**. *Int Immunol* 1998, **10**(8):1217-1227.

46. Schnurch H, Risau W: **Expression of tie-2, a member of a novel family of receptor tyrosine kinases, in the endothelial cell lineage**. *Development* 1993, **119**(3):957-968.

47. Dumont DJ, Yamaguchi TP, Conlon RA, Rossant J, Breitman ML: **tek, a novel tyrosine kinase gene located on mouse chromosome 4, is expressed in endothelial cells and their presumptive precursors**. *Oncogene* 1992, **7**(8):1471-1480.
