# Supplementary figures and images for: Discovering monotonic stemness marker genes from time-series stem cell microarray data
Source: BMC Genomics. 2015 Jan 21;16(Suppl 2):S2. doi: 10.1186/1471-2164-16-S2-S2 (PMC4331716; doi:10.1186/1471-2164-16-S2-S2)

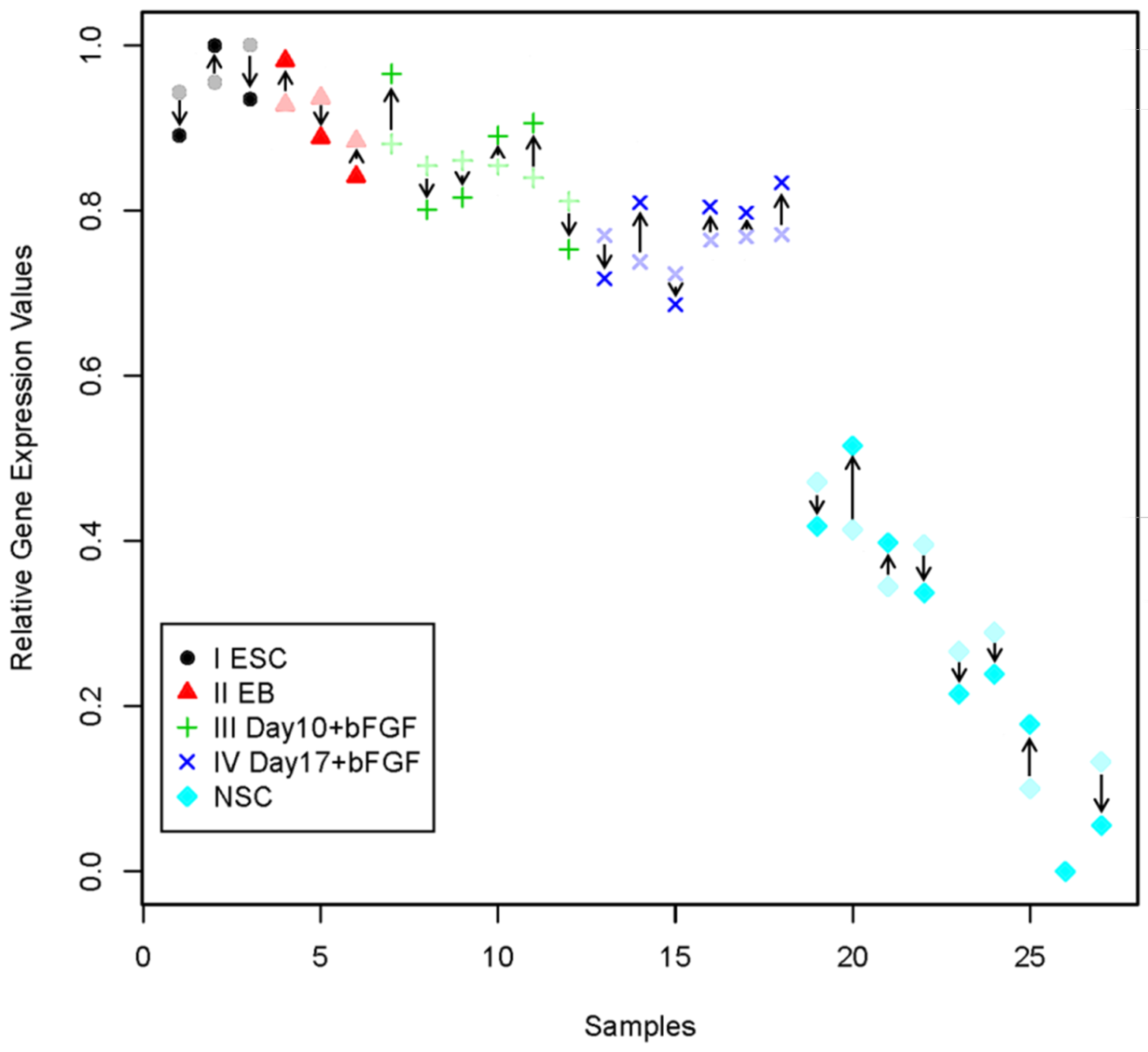

Supplement: Additional file 2 — Figure S1. Illustration of adding noise to original expression values of 223038_s_at (FAM60A) of the ESCN data set. The DEtotal is four after running a simulation with added noise. [file 1471-2164-16-S2-S2-S2.tif]

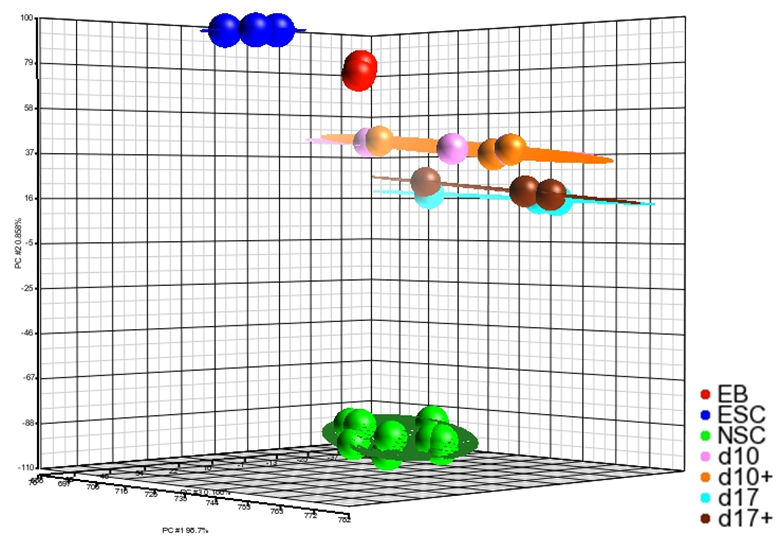

Supplement: Additional file 3 — Figure S2. A three-dimensional scatter plot of the ESCV data set analyzed by principal component analysis. [file 1471-2164-16-S2-S2-S3.tif]

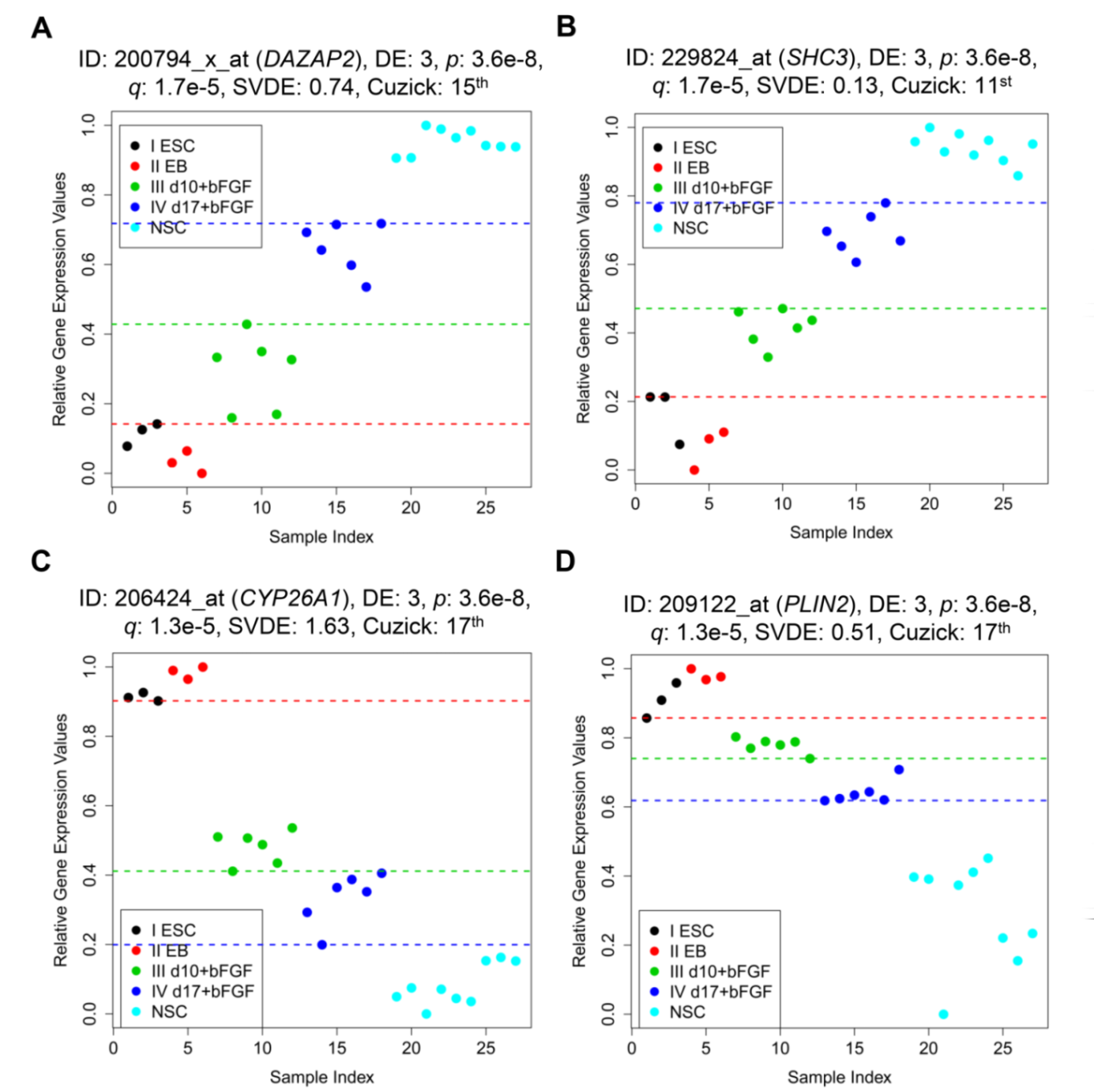

Supplement: Additional file 8 — Figure S3. Scatter plots of the four false positive monotonic genes identified by the Cuzick-test from the ESCN data set. (A) 200794_x_at (DAZAP2); (B) 229824_at (SHC3); (C) 206424_at (CYP26A1); (D) 209122_at (PLIN2). [file 1471-2164-16-S2-S2-S8.tif]

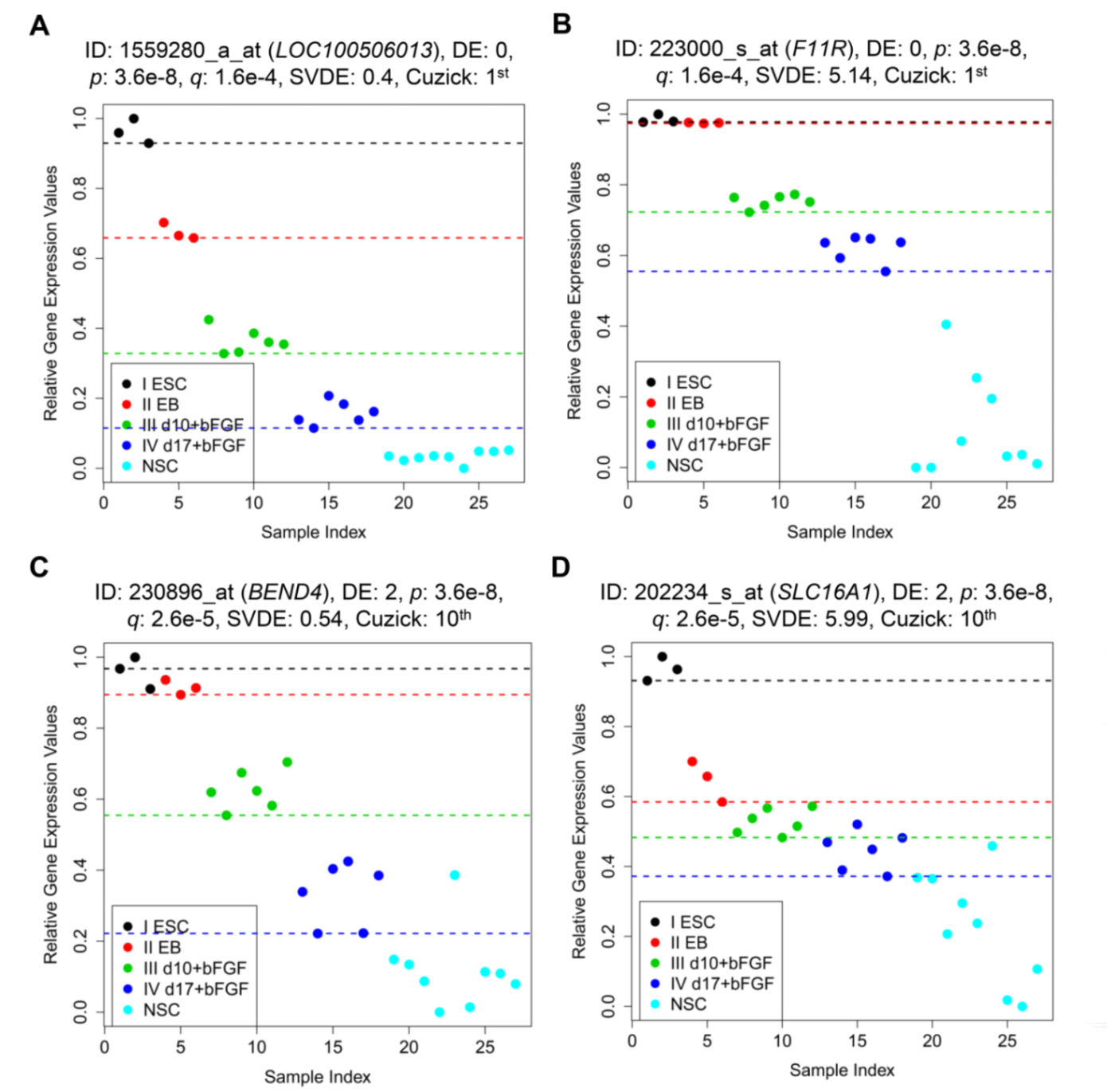

Supplement: Additional file 9 — Figure S4. Scatter plots of the four monotonic genes with the same DEtotal values but different SVDE values identified from the ESCN data set. (A) 1559280_a_at (LOC100506013); (B) 223000_s_at (F11R); (C) 230896_at (BEND4); (D) 202234_s_at (SLC16A1). [file 1471-2164-16-S2-S2-S9.tif]

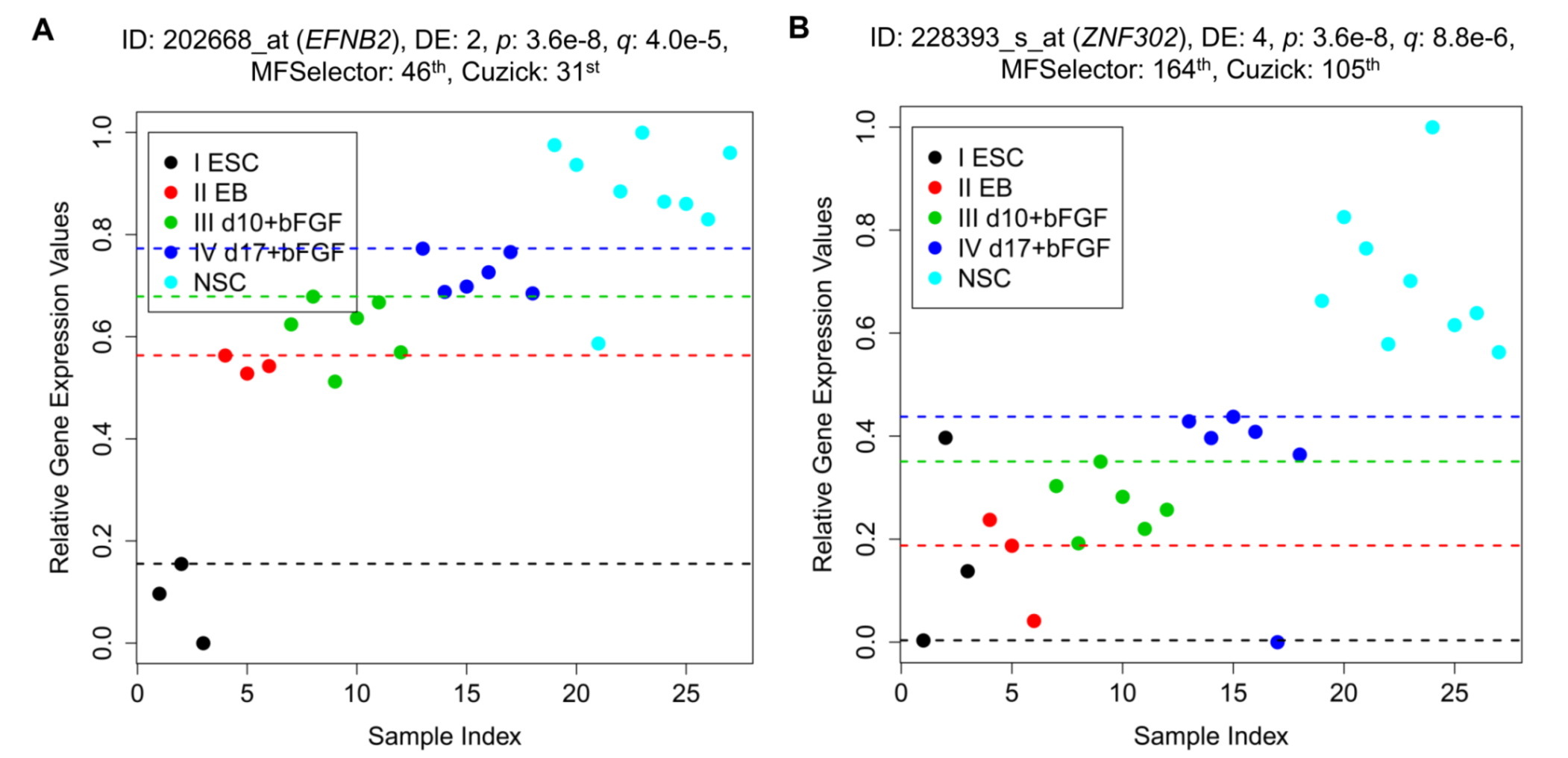

Supplement: Additional file 10 — Figure S5. Scatter plots of the two monotonic genes for the ESCN data set ranked low by the Cuzick-test but high by MFSelector. (A) 202668_at (EFNB2); (B) 228393_s_at (ZNF302). [file 1471-2164-16-S2-S2-S10.tif]

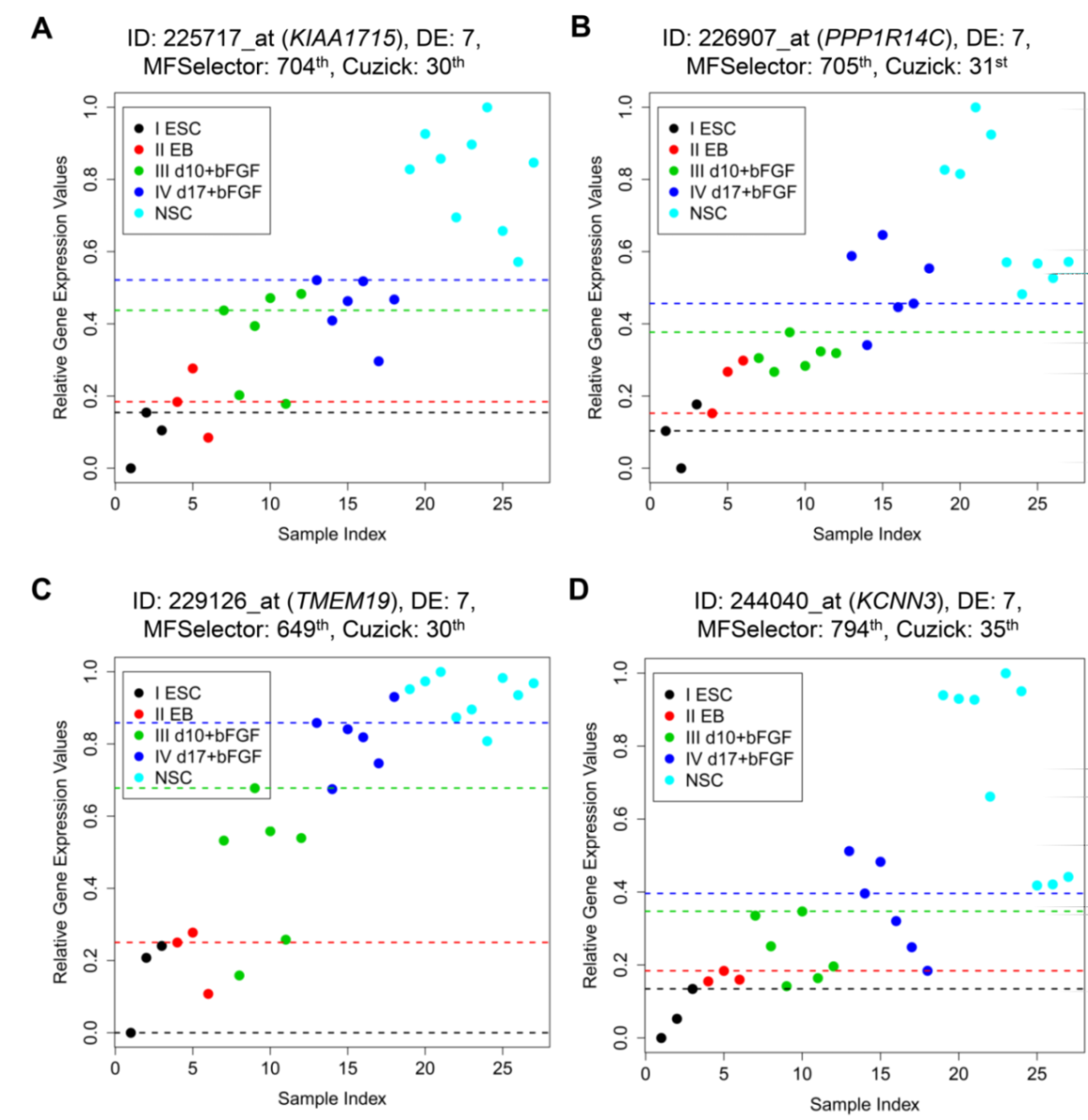

Supplement: Additional file 11 — Figure S6. Scatter plots of the four ascending monotonic genes from the ESCN data set ranked high by the Cuzick-test but low by MFSelector. (A) 225717_at (KIAA1715); (B) 226907_at (PPP1R14C); (C) 229126_at (TMEM19); (D) 244040_at (KCNN3). [file 1471-2164-16-S2-S2-S11.tif]

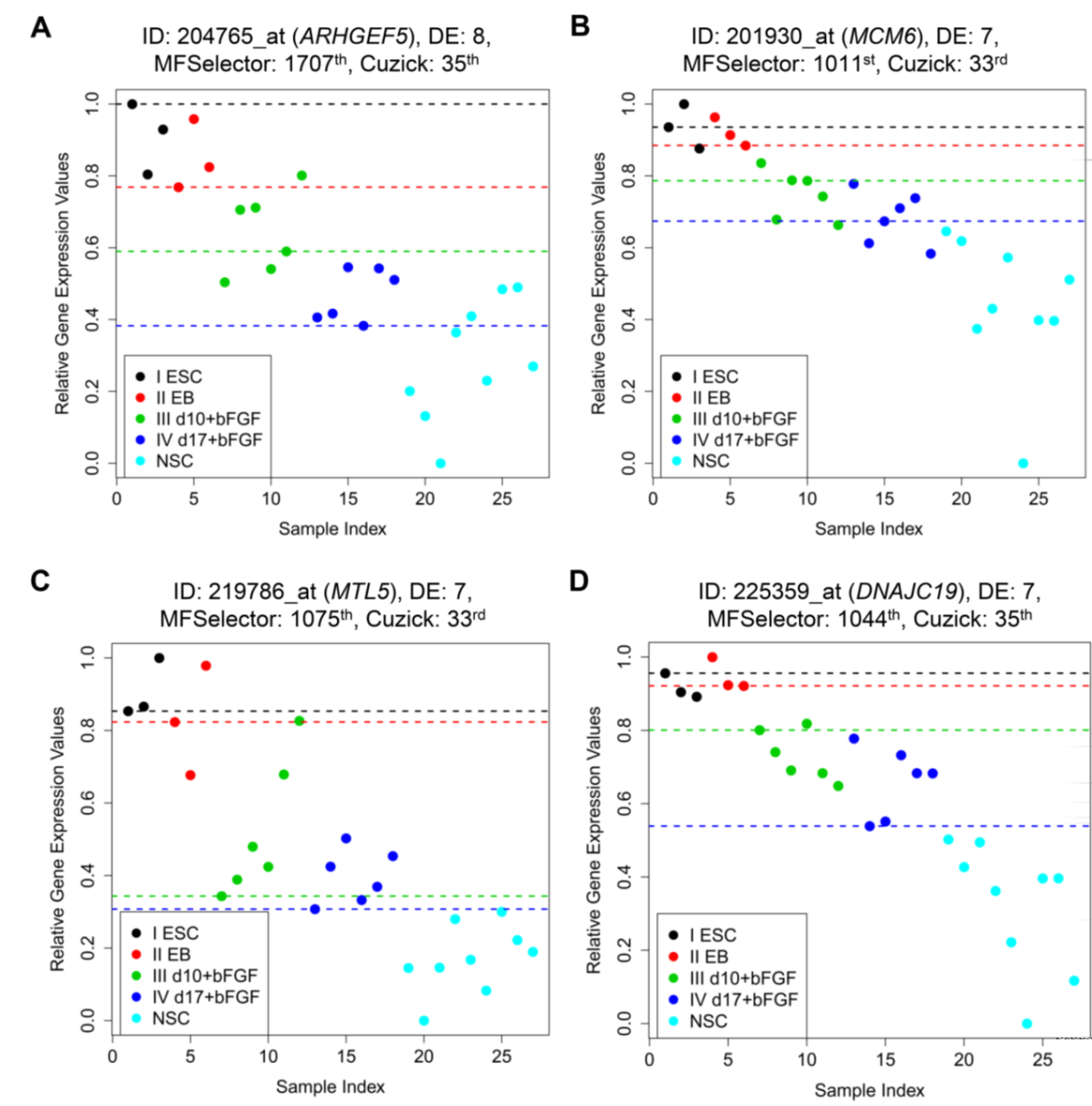

Supplement: Additional file 12 — Figure S7. Scatter plots of the four descending monotonic genes from the ESCN data set ranked high by the Cuzick-test but low by MFSelector. (A) 204765_at (ARHGEF5); (B) 201930_at (MCM6); (C) 219786_at (MTL5); (D) 225359_at (DNAJC19). [file 1471-2164-16-S2-S2-S12.tif]

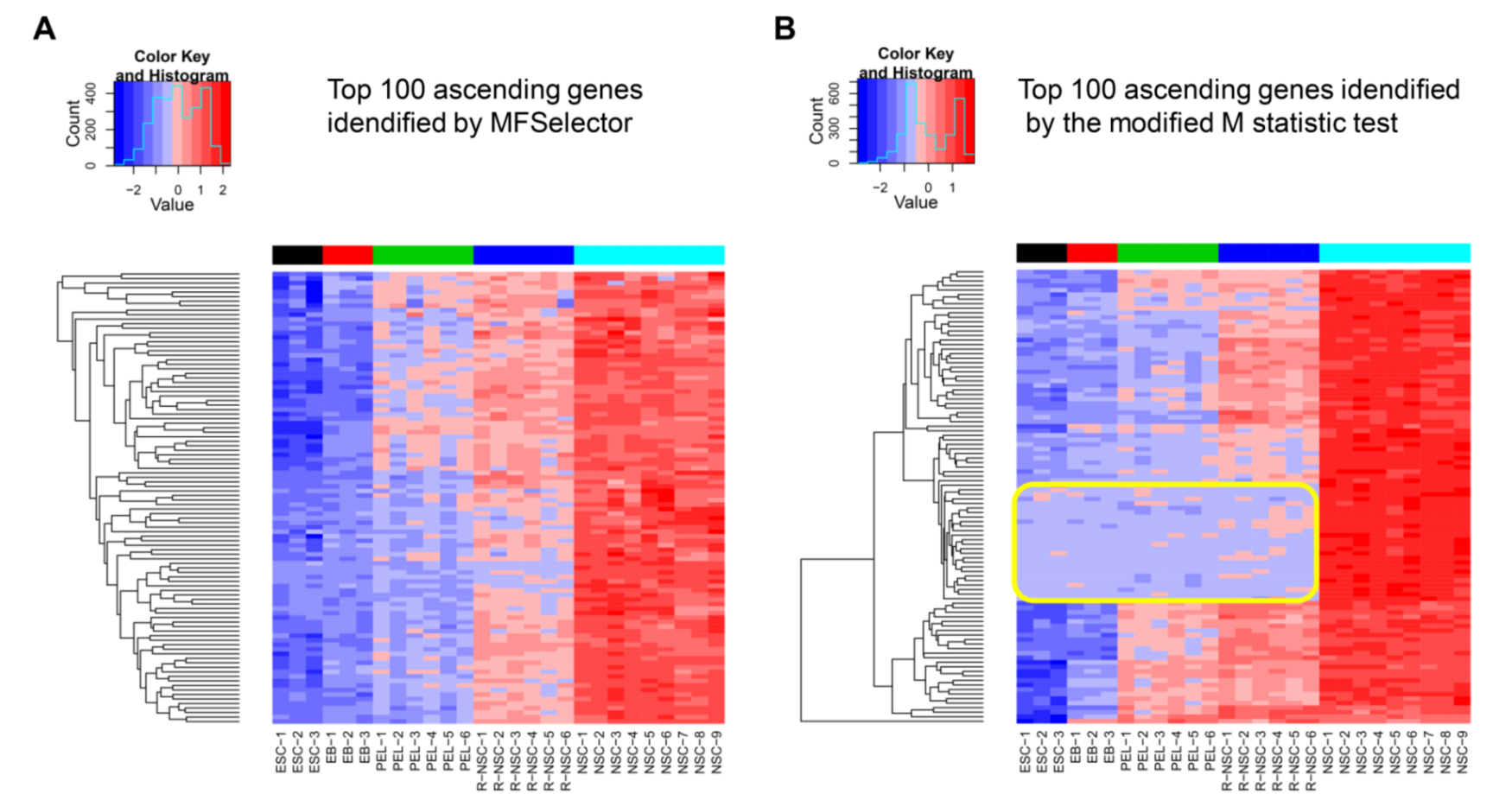

Supplement: Additional file 13 — Figure S8. Heatmaps of the monotonic ascending gene set (the top 100 genes with ascending profiles) of the ESCN data set identified by MFSelector and the modified M statistic test, respectively. (A) The top hundred monotonic ascending gene set chosen by MFSelector; (B) The top hundred ascending gene set chosen by the modified M statistic test. [file 1471-2164-16-S2-S2-S13.tif]

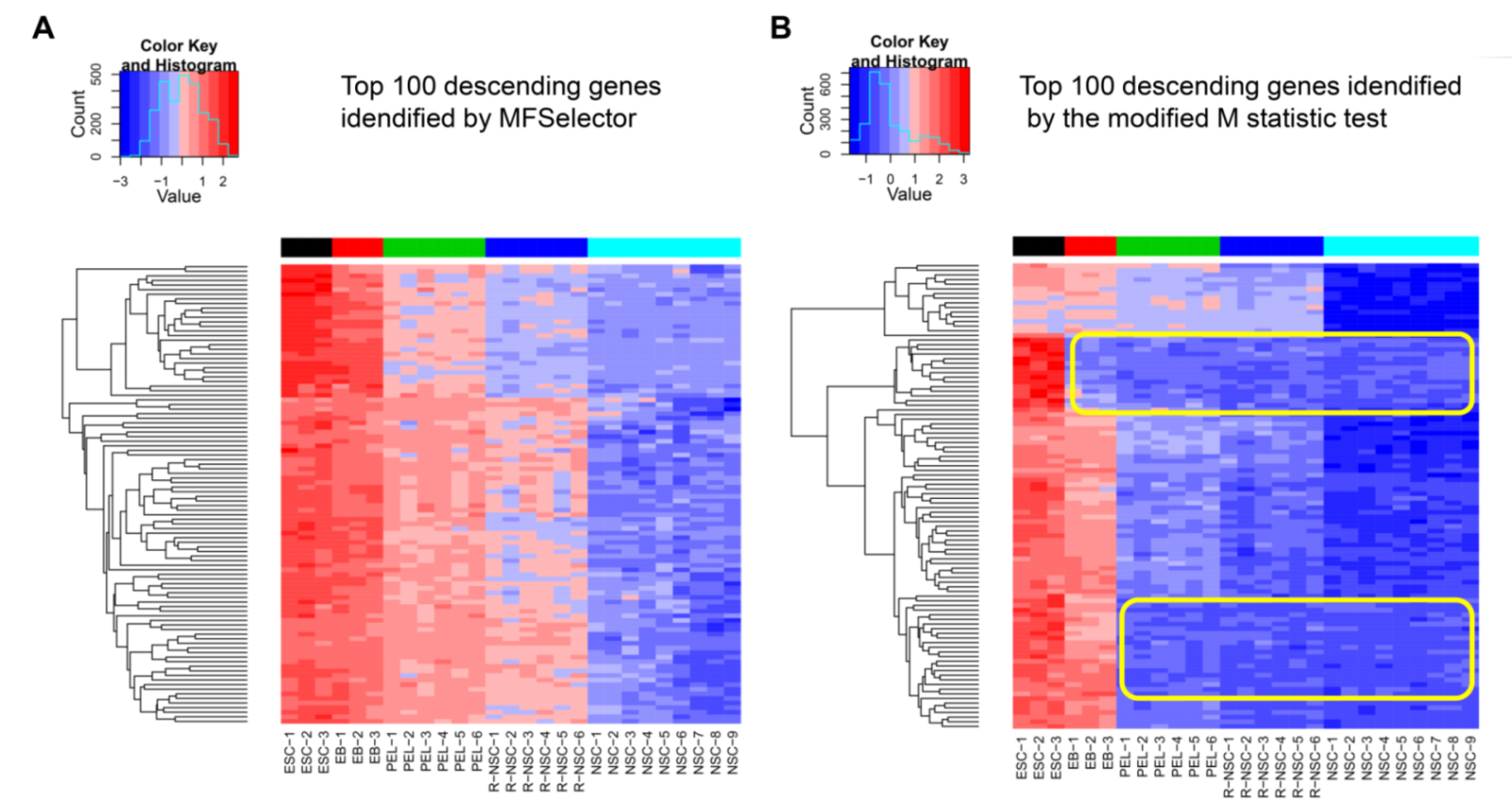

Supplement: Additional file 14 — Figure S9. Heatmaps of the monotonic descending gene set (the top 100 genes with descending profiles) of the ESCN data set identified by MFSelector and the modified M statistic test, respectively. (A) The top hundred monotonic descending gene set chosen by MFSelector; (B) The top hundred descending gene set chosen by the modified M statistic test. [file 1471-2164-16-S2-S2-S14.tif]

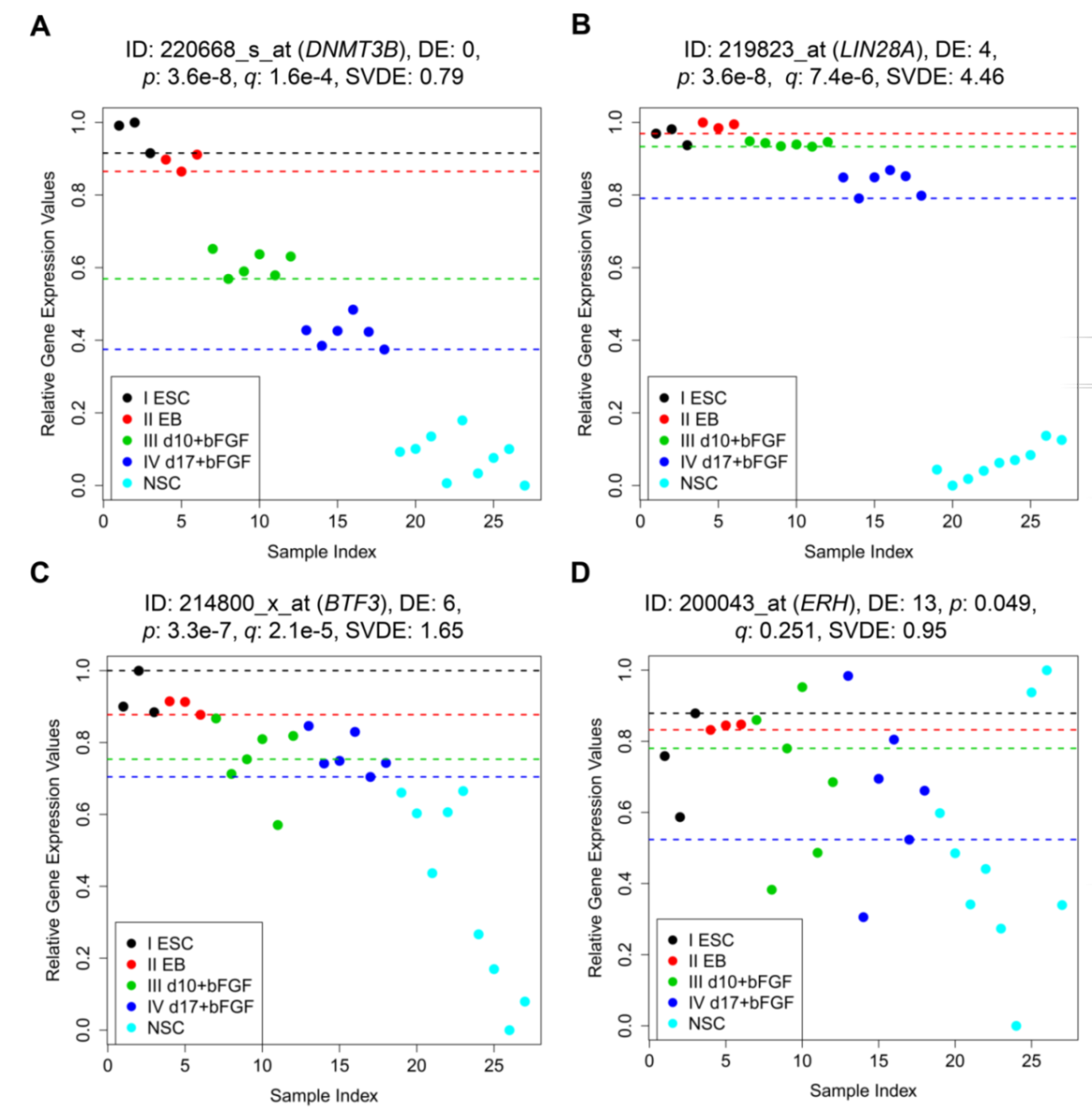

Supplement: Additional file 17 — Figure S10. Scatter plots of the four genes of the ESCN data set reported in the previous study[31] (A) 220668_s_at (DNMT3B); (B) 219823_at (LIN28A); (C) 214800_x_at (BTF3); (D) 200043_at (ERH). [file 1471-2164-16-S2-S2-S17.tif]
